# Effects Of Lake Warming On Behavioural Thermoregulatory Tactics In A Cold-Water Stenothermic Fish

**DOI:** 10.1371/journal.pone.0092514

**Published:** 2014-03-24

**Authors:** Katerine Goyer, Andrea Bertolo, Marc Pépino, Pierre Magnan

**Affiliations:** Centre de Recherche sur les Interactions Bassins Versants – Ecosystèmes Aquatiques (RIVE) Université du Québec à Trois-Rivières, Trois-Rivières, Québec, Canada; Aristotle University of Thessaloniki, Greece

## Abstract

Despite some evidence of within-population phenotypic variation in fish thermal behaviour, the occurrence of alternative tactics of this behaviour is rarely explicitly considered when studying natural populations. Brook charr provide an example of within-population variability in behavioural thermoregulation as revealed by a recent study on a lacustrine population of this species. The objectives of the present study were (i) to determine the influence of natural variability in the lake's thermal profiles on the expression of thermoregulatory tactics, and (ii) to determine the vertical and horizontal movements of individuals at different periods of the day to better understand the spatio-temporal behaviour associated with each thermoregulatory tactic. During summer 2010, 30 adult brook charr were equipped with thermo-sensitive radio transmitters to monitor their selected temperatures and daily movements. These individuals exhibited the same four behavioural thermoregulatory tactics observed in 2003 and 2005, but the expression of two of these was weaker in 2010. This result was associated with lake warming, which constrained the expression of two thermoregulatory tactics: brook charr significantly decreased their selected temperatures and daily movements when the mean daily epilimnion temperature was above 22.4°C. This study shows for the first time that the expression of behavioural thermoregulatory tactics is related to the lake's thermal regime and that the tactics are plastic through time.

## Introduction

In ectotherms, body temperature is largely controlled by environmental temperature, and many mobile organisms have developed thermoregulatory mechanisms to survive and optimize temperature-dependent physiological processes [1],[2],[3]. In fish, behavioural thermoregulation involves the selection of a thermally optimal habitat by means of preference and avoidance responses along thermal gradients [4]. The active selection of a given temperature by fish often leads to daily movements between a feeding area and a zone where temperatures enhance growth efficiency [5]. Since processes such as digestion or growth can differ in their thermal optima [6], these daily movements could provide a bioenergetic advantage by maximizing the rate of different processes occurring at different times [7].

Behavioural thermoregulation has been observed in several salmonid species (e.g. [8],[9]). Being cold-water stenothermic fish, they frequently encounter suboptimal temperatures during the summer and need to seek thermal refuges to maintain a tolerable temperature. Some species, like rainbow trout (*Oncorhynchus mykiss*) and Atlantic salmon (*Salmo salar*), stop feeding and defending their territory to seek cool-water sites when the temperature is over a specific threshold [10],[11]. This behaviour is also observed in brook charr (*Salvelinus fontinalis*) when water temperatures reach 20 to 23°C [12],[13]. In the absence of thermal refuges, Robinson et al. [14] observed that variable interannual summer water temperatures strongly influenced brook charr behaviour and life history: exposure to chronically stressful summer temperatures (>20°C) resulted in decreased growth, feeding, and reproduction rates, and increased mortality.

It has long been believed that species have only one “final thermal preferendum” (i.e., the temperature actively selected independently of previous thermal experience; *sensu* [15]), suggesting that thermal behaviour should be similar among conspecifics. However, a large number of experimental studies have shown that temperature selection can be influenced by factors such as acclimation [16], sex [17], intra- and inter-specific interactions [18], and feeding [19], therefore challenging the general validity of this paradigm. Despite these examples of within-population phenotypic variation in thermal behaviour, the occurrence of alternative thermoregulatory tactics is still rarely explicitly considered when studying natural populations (e.g. [20]).

A recent study revealed the existence of four behavioural thermoregulatory tactics within a lacustrine brook charr population [1]. These tactics were defined relative to the observed periodic variations in the selected temperature of individuals: one tactic was characterized by a temporal pattern of diel (ca. 24 h), crepuscular (ca. 12 h), and finer periodicities (ca. 8 h), hereafter “tactic I”; another tactic was characterized by broad-scale (>24 h), crepuscular, and finer periodicities, hereafter “tactic II”; the third was characterized only by a broad-scale periodicity, hereafter “tactic III”; and the fourth was characterized by a diel periodicity only, hereafter “tactic IV” [1]. Individuals displaying the tactics I and II had mean body temperatures approximately 2°C higher than those displaying tactics III and IV [1]. Whereas these tactics existed along a gradient of thermal behaviours in terms of amplitude, periodicity, and mean selected temperature [1], grouping the individuals in such way put emphasis on the within-population plasticity in behavioural thermoregulation.

Based on the observed selected temperatures and radio-tracking data, Bertolo et al. [1] suggested that (i) the expression of crepuscular and finer periodicities is associated with individuals performing short excursions into the epilimnion (i.e., the warmest, well-mixed surface layer of a stratified lake), and (ii) the expression of diel vs. broad-scale thermal periodicities is associated with variations in the horizontal movements of individuals. Some studies have documented the existence of a resource polymorphism among brook charr of this population, with a pelagic ecotype feeding on zooplankton and a littoral ecotype feeding on benthic organisms [21],[22],[23]. Such within-population variability in both prey and habitat use could have led to the development of alternative thermal behaviours. Polymorphism was therefore hypothesized to be the mechanism behind the presence of distinct behavioural thermoregulatory tactics [1]. The first objective of this study was to determine the influence of the lake's thermal regime on brook charr thermoregulatory tactics by studying the thermal behaviour of individuals in the contrasting conditions of the lake's thermal regime (original data from the present study coupled with data from [1]). More specifically, we predicted that the expression of tactics associated with short excursions into the epilimnion (i.e., tactics I and II) would be weaker when surface temperature approaches the tolerance threshold of the species. Brook charr is a cold-water stenothermic species that tends to avoid temperatures higher than 20°C [13],. Warmer thermal profiles should therefore limit fish movements towards epilimnetic habitats. The second objective of the study was to determine the vertical and horizontal movements of individuals using radio-tracking at different periods of the day (dawn, day, dusk, and night) to better understand the spatio-temporal behaviour associated with each thermoregulatory tactic.

## Materials And Methods

### Study Lake

The study was carried out from 5 July to 29 August 2010 in Lake Ledoux (46° 38′ N, 73° 15′ W), Mastigouche Reserve, Québec, Canada. Data from the study conducted during the summers 2003 and 2005 on Lake Ledoux [1] were also included in the analysis. Lake Ledoux is a typical small oligotrophic temperate zone lake with respect to surface area (11.9 ha), mean depth (5.5 m), maximum depth (17.0 m), and general physicochemical characteristics [26]. The summer stratification of the lake provides a heterogeneous thermal habitat (ranging from 5.5 to 27.3°C), and thus favourable conditions for fish behavioural thermoregulation. Brook charr is the only fish species in the lake, and sport fishing is rigorously controlled by the Québec Government [26]. The lake was closed to fishing during the study. Our access to lake Ledoux was approved by the Ministère du Développement durable, de l'Environnement et des Parcs (SEG: 2010-05-20-046-04-S-P).

### Thermo-Sensitive Radio Transmitter Implantation

Fish were captured in June 2010 with Alaska traps covering the littoral (<2 m depth) and deeper (>4 m depth) zones of the lake. Thirty adult individuals (>180 g) were equipped with 3.6 g radio transmitters (model ATS-F1540, Advanced Telemetry System [ATS], Isanti, MN, USA) using surgical procedures adapted from Bélanger and Rodríguez [27]. The fish were anaesthetized with eugenol (clove oil; 50 mg/L) and placed dorsal side down on a V-shaped surgical board covered with synthetic foam soaked in Aquarium Pharmaceuticals Stress coat to reduce mucus loss. Constant gill irrigation containing a dilute anaesthetic solution (clove oil; 20 mg/L) kept the fish sedated while maintaining a regular rate of opercular beating [28]. Transmitters were implanted in the peritoneal cavity through a small incision made on the ventral side in front of the pelvic fins. The transmitter antenna was guided through a hole pierced between the anal and the pelvic fins. To reduce the risk of infection, oxytetracycline (50 mg/kg of fish mass) was injected into the peritoneal cavity before closing the incision with three stitches of non-absorbable synthetic monofilament (Ethilon noir 4/0+FS2; CDMV, St-Hyacinthe, QC, Canada). Transmitter implantation took approximately four minutes. Fish were then kept in an enclosure (3 m×4 m×5.5 m depth) for two to four days. All fish released into the lake were in apparently good shape and behaved normally.

Whereas external radio transmitters (model ATS-F1970) were used in 2003/2005, we used internal radio transmitters in 2010. A preliminary controlled experiment showed that both transmitters achieved ambient temperature within approximately 20 minutes (K. Goyer, personal observation). Since this delay was considered negligible compared to the finer temporal scale resolution of the analyses (i.e., 4 h; see details below), we consider that data from 2003, 2005, and 2010 are comparable.

### Temperature Data

The lake's temperature profile was measured by 21 thermographs (iBcod, Alpha Mach Incorporation, Mont St-Hilaire, QC, Canada) moored at 0.5 m intervals from the surface to 10 m in depth. Temperature data were recorded each hour from 5 July to 29 August 2010 (Fig. S1). Thermographs in the upper 4 m were protected by perforated white plastic tubes to prevent warming from solar radiance.

Two radio receivers (ATS models R2100 and R4500) were installed on two rafts anchored approximately 400 m apart to cover the largest area of the lake. Each receiver was connected to two loop antennas oriented perpendicularly and to a data logger (ATS-Data Collection computer models D5041 and R4500) that recorded the temperature of each transmitter every 30 minutes. Given the high percentage of missing data for some fish, only 16 individuals with less than 35% of missing data were retained for the analyses. These fish had a mean fork length of 314 mm (range: 291–374 mm) and weighed 310 g on average (range: 223–480 g).

### Radio-Tracking

Individual fish were located four times a day (dawn, day, dusk, and night) and four days a week from 5 July to 13 August 2010. Dawn and dusk recordings were made within 2 h of sunrise and sunset, respectively, while day and night recordings were within 2 h of noon or midnight. Each fish was located in a random order from an electric-powered boat using a radio receiver (ATS model R2000) equipped with a loop antenna. For each location, we recorded the geographic coordinates (UTM, North American Datum 1983) using a global positioning system (hand-held Garmin GPSMAP 76, Olathe, KN, USA) equipped with a wide area augmentation system (WAAS).

### Statistical Analyses

#### Thermal Tactics

Temperature time series were analyzed for each fish from their mean hourly temperatures from 5 to 23 July 2010. A malfunction of one of the two receivers after this date did not allow us to extend the analyses until the end of the sampling period. Temperature time series were modelled with asymmetric eigenvector maps (AEM; [29],[30]) to determine which temporal scales are relevant in the patterns of brook charr temperature selection. We preferred this approach to Fourier analysis or harmonic regression because of its flexibility in responding to missing values. AEM and principal coordinates of neighbour matrices (PCNM; [31]) are two approaches that give equivalent results in modelling patterns in one dimension, such as time series (P. Legendre, Université de Montréal, personal communication). AEM analysis first creates a series of sinusoidals ([32]; hereafter called AEM) whose periods are measured in hours and decrease progressively, with the largest corresponding to the study period (450 h) and the smallest to approximately 4 h. AEM were then used as independent variables in multiple regressions to model temperature data for each individual separately [29],[31]. To facilitate comparisons among individuals, temperature data from all individuals were modelled with the same set of AEM that was created from a unique sampling grid (one datum per hour). Only AEM having a positive and significant (α = 0.05) index of autocorrelation (Moran's I [33]) were kept for analyses. To control for artificial collinearity among AEM occurring when data are missing [34], we measured the collinearity between independent variables using the variance inflation factor (VIF), and we progressively removed AEM having a VIF greater than 10 [35]. AEM were built using the AEM package [29] in the R statistical language [36].

**Figure 1:**
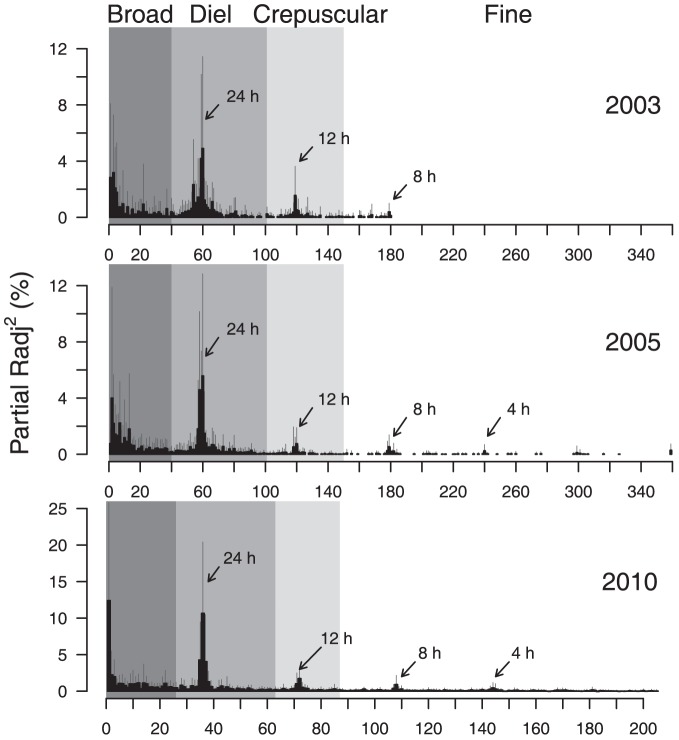
Scalogram based on the average partial R_adj_^2^ distribution in 2003/2005 (PCNM data) and 2010 (AEM) data. PCNM and AEM periodicities gradually decrease from left to right. Error bars represent the standard deviation of partial R_adj_^2^ among individuals. Arrows indicate approximate PCNM and AEM periodicities. Grey bands are the boundaries of the four temporal scales used in PCA analysis. Panels for 2003 and 2005 are redrawn from [1].

The relative importance of each AEM for each individual was assessed by its contribution to the adjusted R^2^ of the model (hereafter called partial R_adj_^2^ [1]). This importance can be represented at the population level in a scalogram, where the partial R_adj_^2^ values, averaged among all individuals (± SD), are plotted against each AEM, which are ranked by decreasing period. According to the scalogram shape (Fig. 1) and to the results obtained by Bertolo et al. [1], AEM were grouped into four temporal scales: “broad” (periodicity >35 h; AEM 1–25), “diel” (periodicity 35–14 h; AEM 26–62), “crepuscular” (periodicity 14–10 h; AEM 63–87), and “fine” (periodicity <10 h; AEM 88–205). A principal component analysis (PCA) was performed on Hellingher-transformed [37] cumulative partial R_adj_^2^ for the four temporal scales computed for each individual. Whereas the scalogram helped to identify the relevant periodicities at the population level, we used the PCA to highlight the presence of different thermal tactics at the within-population level and to identify the relative importance of each temporal scale for each individual. For each thermal tactic, the mean individual selected temperatures were compared between periods of the day and thermal conditions by two-way ANOVA.

#### Daily Movements

While not used to define the tactics themselves, fish daily movements were used to better interpret the spatio-temporal behavioural patterns associated with each thermoregulatory tactic defined above. Fish daily movements were decomposed into two parts: the frequency of their daily excursions into the epilimnion and their horizontal movements. The frequency of daily excursions into the epilimnion was defined as the number of times a fish was found in the epilimnion relative to the total number of positions recorded for that fish during the day (expressed in percent of locations in the epilimnion per day). To facilitate inter-annual comparisons, we analyzed data that fell within the same temporal window for the three years. Thus, the frequency of daily excursions into the epilimnion was computed from data recorded between 14 July and 29 August (2003 and 2005 from Bertolo et al. [1]; 2010 from this study). We used a regression tree analysis [38] to model the frequency of daily excursions into the epilimnion with the mean daily epilimnion and metalimnion (i.e., the portion of the water column below the epilimnion characterized by a temperature gradient >1°C/m depth) temperatures, day of the year, and year as explanatory variables. The tree was built using the rpart package [39] in the R statistical language [36]. Graphically, trees are represented by a dendritic network in which the proportion of the total sum of squares explained by each split (expressed as the r^2^ value) is represented by the lengths of the vertical lines [40],[41]. To reduce excessive splitting and data overfitting, the regression tree was pruned to an optimal size using 10-fold cross-validation [40] and the 1-SE rule, which favours the smallest tree for which the cross-validated error falls within one standard error of the minimum relative error determined by cross-validation [38],[39]. According to De'ath and Fabricius [40], *V*-fold cross-validation based on repeated observations of the sampling units (here individual fish) can lead to optimistic predictions of error rates and overestimations of the best tree size, since subsamples within units (here frequencies of daily excursions for the same fish) are likely to be correlated. To overcome this problem, De'ath and Fabricius [40] suggested that only whole sampling units (here all frequencies of daily excursions for the same fish) be selected in the subsets so that units are predicted only from other units. We therefore applied this modification to the 10-fold cross-validation by creating 10 subsets, each one of them containing only complete sampling units. We tested the pruned tree with a permutation method to determine whether it explained significantly more variance than a random regression tree of equal complexity [41].

We investigated the influence of water temperature on the horizontal movements of fish by comparing the mean distance travelled by each individual at each period of the day above and below a mean daily epilimnion temperature threshold (identified in the regression tree analysis; see Results section). The travelled distance was defined as the linear distance between two consecutive locations. For each thermal tactic identified in the time series analyses (see Results section), the distances were compared between periods of the day and thermal conditions by two-way ANOVA. All analyses were performed using the R statistical language [36].

### Ethics Statement

This study was approved by the Animal Care Committee of the University of Québec at Trois-Rivières (Comité de Bons Soins aux Animaux de l'UQTR – CBSA; certificate #2010-P.M.26).

## Results

### Thermal Tactics

Lake Ledoux was stratified during the whole study period (5 July to 29 August 2010; Fig S1). The epilimnion and metalimnion were estimated daily based on the lake's thermal profiles (*sensu* [42]); their mean temperatures (± SD) were 22.6±1.9°C and 13.1±0.8°C, respectively. Over this period, the metalimnion started at depths ranging from 1.5 to 4 m while its lower limit was stable through the study period at 8.5 m depth. Temperatures for the period considered in the thermal tactics analyses (5–23 July) were significantly warmer in 2010 than in 2003 and 2005 (Table 1).

**Table 1:** Mean epilimnion and metalimnion temperatures (±SD; °C) for the periods considered in the thermal tactics analyses.

Year	Epilimnion	Metalimnion
2003	22.6±1.3 a	10.7±1.2 a
2005	23.3±1.3 a	10.9±0.8 a
2010	24.9±1.2 b	14.0±0.6 b

Means with different letters are significantly different among years, as determined by an ANOVA (or a Kruskal-Wallis test when the assumptions of ANOVA were not met) followed by an a posteriori Tukey comparison test (P<0.05).

Temperature data modelled by multiple regressions using AEM contained between 9% and 35% of missing values per fish. Nevertheless, individual thermal patterns were well fitted by AEM, with a mean adjusted coefficient of determination (R_adj_^2^) of 0.75±0.08 (Fig. S2). At the population level, relevant periodicities in thermal behaviour identified by the scalogram were strikingly similar to those revealed by Bertolo et al. [1] (Fig. 1). The highest mean partial R_adj_^2^ was observed for the first AEM, which indicates the presence of a broadscale thermal pattern. A signal of similar magnitude was found around AEM 36, which corresponds to a diel periodicity. Weaker signals were also observed at finer temporal scales (AEM 72, 108, and 144), corresponding to periodicities of 12, 8, and 4 hours, respectively (Fig. 1). These periodicities were used to define the four temporal scales described in the Materials and Methods section and that are essentially the same as those used in Bertolo et al. [1].

**Figure 2:**
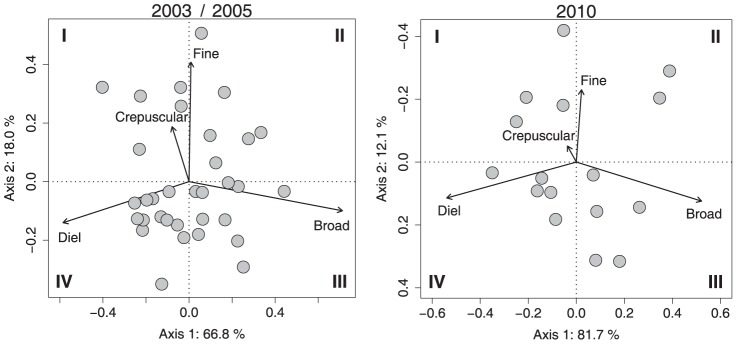
PCA on the cumulative partial R_adj_^2^ of the 2003/2005 and 2010 data relative to the four temporal scales identified in Bertolo et al. (2011) [1]: broad, diel, crepuscular, and fine. Data from 2010 are compared to 2003/2005. Left panel was redrawn from [1].

PCA conducted on cumulative partial R_adj_^2^ relative to the four temporal scales confirmed the pattern identified in 2003/2005 (Fig. 2). The two first axes are strongly correlated to the temporal scales defined in the scalogram (Fig. 1) and were arbitrarily used to operationally define thermal tactics associated with the four quarters of the PCA plot [1]. This classification describes a gradient of thermal behaviours within the population ranging from diel to broad periodicities according to the first axis and from absence to presence of finer periodicities (i.e., crepuscular and fine) according to the second axis. The first axis explained a larger portion of the variation than in 2003/2005 (81.7% versus 66.8%); individuals with negative scores on the first axis showed thermal patterns characterized by clear diel cycles while those with positive scores were related to thermal patterns characterized by broad temporal scales. Whereas the second axis in 2003/2005 was associated with a 2°C temperature gradient in the mean body temperature of individuals [1], we did not find any difference in the mean body temperature in 2010 along this axis. Even though this axis was characterized by both crepuscular and fine periodicities in both 2003/2005 and 2010, it explained relatively less variation in 2010 (12.1%) than in 2003/2005 (18.0%). Based on these results, our subsequent analyses focussed on the first axis of the PCA for defining the tactics in 2010. Thus, our subsequent analyses pooled tactics I and IV as defined in Bertolo et al. [1] into the “I/IV” tactic whereas their tactics II and III were pooled in the “II/III” tactic (see Fig. 2). Such a classification allows us not only to make a direct comparison with the Bertolo et al (2011) results, but also makes conceptualization of the within-population plasticity of thermal behaviour more straightforward.

### Vertical And Horizontal Daily Movements

**Figure 3:**
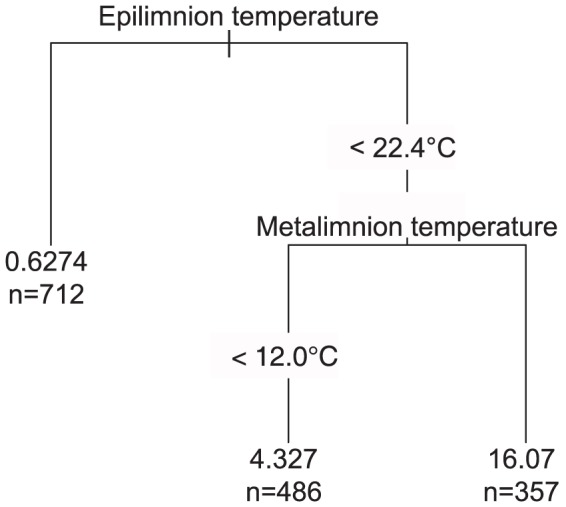
Regression tree model of daily excursion frequencies into the epilimnion based on mean daily epilimnion and metalimnion temperatures, day of the year, and year (data from 2003, 2005, and 2010). Each split is labelled with the variable and the value determining the split. The tree was pruned to a size of three terminal groups (branches), each one labelled with the mean percentage of daily excursions and the number of observations within the group. The tree explained 15% of the total variation (P<0.05). The vertical length of each split is proportional to the explained variation. Data were collected during the same period (14 July to 29 August) in the three study years.

The regression tree was pruned to a size of three branches and explained a relatively low but significant part of the variation of daily excursion frequencies into the epilimnion (r^2^ = 0.15, P<0.05; Fig. 3). The results indicate that the frequencies of these excursions are correlated with both the mean daily epilimnion and metalimnion temperatures. The fish first responded to the epilimnion's thermal conditions (r^2^ = 0.08), making almost no excursions into this layer when the mean daily temperature exceeded 22.4°C. This threshold was used to compare the behaviour of brook trout under different thermal regimes in subsequent analyses. When the epilimnion was colder than the threshold value of 22.4°C, the mean daily metalimnion temperature also affected fish behaviour (r^2^ = 0.07), although in this case, warmer temperatures (>12.0°C) promoted excursions into the epilimnion.

**Figure 4:**
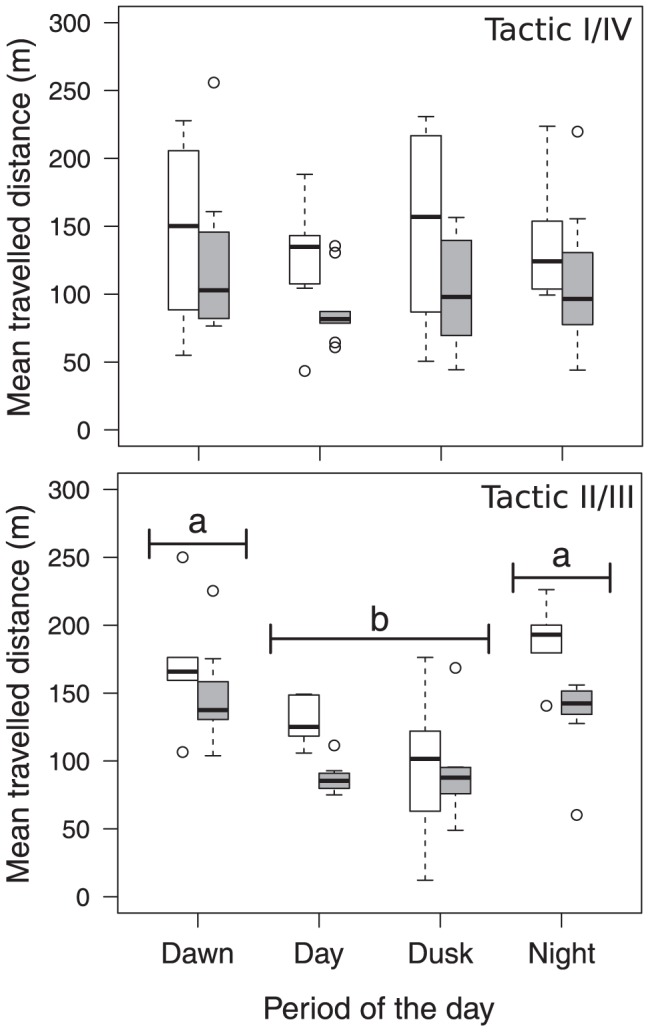
Box plots showing the mean horizontal distances travelled by fish displaying the I/IV (n = 9) and II/III (n = 7) tactics for each period of the day (2010 data only). Distances presented for each period correspond to the distance travelled between this period and the previous one. Bar colour: distance travelled when the mean daily epilimnion temperature was below (white) or above (grey) 22.4°C. The box plots show median values with the 25^th^ and 75^th^ percentiles, the whiskers show the range of values falling within 1.5 interquartile ranges of either quartile, and the circles represent outliers. Periods with the same letter are not significantly different (post-hoc Tukey test, P<0.05).

The horizontal distances travelled by fish varied from 53 to 1580 m (mean ± SD; 466±264 m) per day and from 2 to 663 m (mean ± SD; 122±112 m) between two consecutive location periods. For both the I/IV and II/III thermal tactics, the extent of horizontal movements significantly decreased when the mean daily epilimnion temperature exceeded the thermal threshold of 22.4°C, which was determined in the regression tree analysis (I/IV tactic: F_1,61_ = 6.66, P<0.05; II/III tactic: F_1,42_ = 7.29, P<0.01; Fig. 4). Even though the horizontal distances travelled by individuals of both tactics were not significantly different (F_1,56_ = 1.29, P>0.05), daily patterns differed between the two tactics. Whereas individuals displaying the I/IV tactic showed no significant differences in the horizontal distances they travelled at different periods of the day (F_3,61_ = 0.51, P>0.05; Fig. 4), individuals from the II/III tactic were significantly more mobile during the night and at dawn than during the day and at dusk (F_3,42_ = 10.34, P<0.001; Fig. 4). Daily patterns were not affected by the epilimnion temperature for either tactic (Fig. 4).

### Selected Temperatures

**Figure 5:**
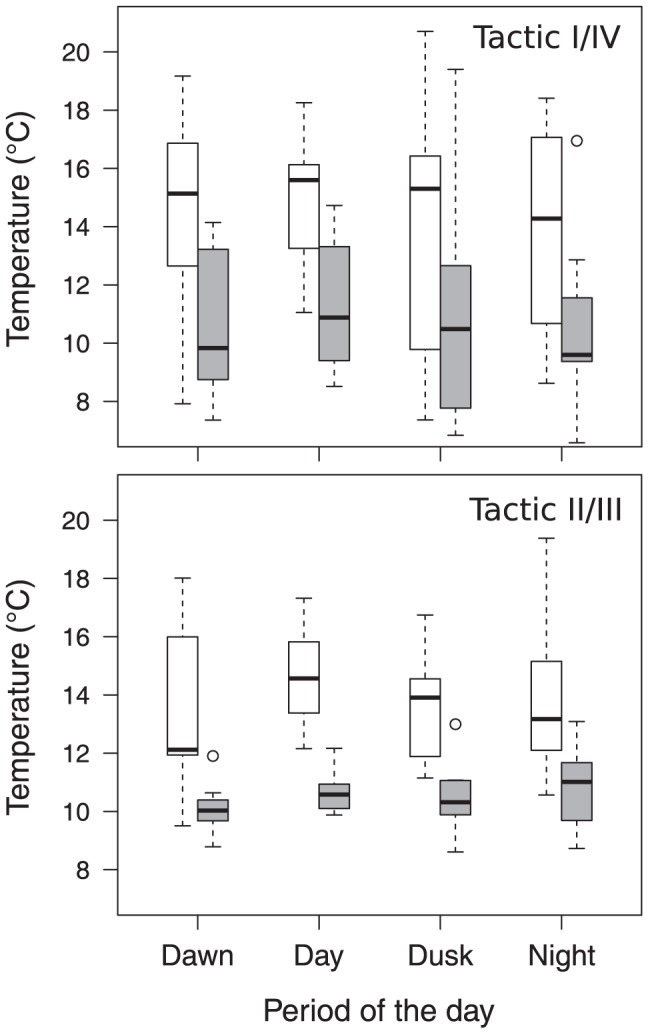
Box plots showing the mean selected temperatures for individuals displaying the I/IV (n = 9) and II/III (n = 7) tactics for each period of the day (2010 data only). Bar colour: distance travelled when the mean daily epilimnion temperature was below (white) or above (grey) 22.4°C. The box plots show median values with the 25^th^ and 75^th^ percentiles, the whiskers show the range of values falling within 1.5 interquartile ranges of either quartile, and the circles represent outliers.

Mean individual body temperatures significantly decreased with an increase of epilimnion temperature for both tactics (I/IV tactic: F_1,64_ = 15.49, P<0.001; II/III tactic: F_1,48_ = 40.44, P<0.001; Fig. 5). Temperatures selected by I/IV and II/III tactics were not significantly different (F_1,56_ = 0.61, P>0.05), varying between 9.1 and 18.1°C (mean ± SD; 14.0±3.1°C) when the mean daily epilimnion temperature was below 22.4°C and dropping to 7.5 to 16.0°C (mean ± SD; 10.8±2.3°C) when it was higher than 22.4°C. Individuals from both tactics did not differ in their diel pattern of selected temperatures (I/IV tactic: F_3,64_ = 0.23, P>0.05; II/III tactic: F_3,48_ = 0.42, P>0.05; Fig. 5). As for the mean travelled distances (Fig. 4), fish displaying the II/III tactic exhibited a lower variability in their selected temperatures than those from the I/IV tactic (Fig. 5).

## Discussion

### Thermal Tactics

Our results confirm the existence of alternative behavioural thermoregulatory tactics previously observed within the same fish population [1], suggesting that these tactics persist through time. Although we found strikingly similar patterns in 2010 and in 2003/2005 in terms of periodicity among the four tactics (Figs. 1 and 2), our results indicate that the expression of these tactics is related to the lake's thermal regime. In contrast to Bertolo et al. [1], we did not observe significant differences in the mean body temperature among tactics in 2010, and the expression of the tactics along the second axis was weaker than in 2003/2005. This result is probably due to the substantial difference in the lake's thermal conditions among the three sampling periods (Table 1). Even though the average thermal regimes of the lake were relatively similar for the three study years (our Fig. S1; Figs. S1 and S11 in [1]), thermal periodicities were assessed at warmer temperatures in 2010 than in either 2003 or 2005. In the two earlier years, both the mean epilimnion and mean metalimnion temperatures were significantly lower than in 2010 (by 2.3 and 3.3°C and 1.6 and 3.1°C, respectively). Because brook charr is a cold-water stenotherm [24], such increases in water temperature could lead to behavioural thermoregulatory changes. The analysis of both vertical and horizontal daily movements offers independent support for this hypothesis (see discussion below).

### Daily Vertical Movements

Our results revealed that the frequency of daily excursions of both the I/IV and II/III tactics into the epilimnion significantly decreased when this zone exceeded 22.4°C. This behaviour has already been observed in lake charr (*Salvelinus namaycush*): Snucins and Gunn [43] found that fish movements into the epilimnion, assumed to be feeding forays, were low (3% of all observations) and brief when the water temperature in this layer rose above their upper thermal tolerance. Other studies conducted over a large range of latitudes have shown that brook charr change their behaviour by migrating to cooler upstream reaches in streams [25] or to deeper water in lakes [44], or by selecting cool-water sites [12],[13] when water temperatures reach 20 to 23°C. These limiting temperatures are consistent with the one observed in our study (22.4°C) and can be considered as a threshold over which temperatures are avoided by our brook charr population under natural conditions. However, this threshold could probably vary depending on the spatial context in which the population evolved [45]. Although field observations have shown that brook charr can tolerate temperatures of 24 to 26.5°C [46], several studies provided evidence that exposure to temperatures above 20°C can lead to negative effects on metabolism, growth, feeding, and mortality [14],[25].

Our results also suggest that the forays were favoured by a relatively cool epilimnion (i.e., <22.4°C) when thermal differences between this layer and the metalimnion were reduced. In fact, the frequency of excursions into the epilimnion was maximal when the epilimnion temperature was below 22.4°C and the metalimnion temperature above 12.0°C. This could be because the relatively low temperature of the epilimnion does not stimulate individuals to seek a cold thermal refuge (grey bars in Fig. 5), allowing them to benefit from the near-optimal thermal conditions of the metalimnion. In most fish species, growth increases with temperature until an optimum, beyond which it decreases [47]. Since the optimal growth temperature for brook charr is approximately 14.6°C [48], such an increase in the metalimnion temperature could lead to enhanced efficiencies of physiological processes including feeding, digestion, and growth rates [47]. Much brook charr feeding occurs in the epilimnion, either in the littoral zone to feed on benthic organisms or in the pelagic zone to feed on zooplankton [22]. More excursions into epilimnion would therefore maximize growth efficiency by increasing feeding opportunities. A relatively cool epilimnion coupled with near-optimal temperatures in the metalimnion might thus have a positive synergistic effect on brook charr growth.

### Daily Horizontal Movements

**Figure 6:**
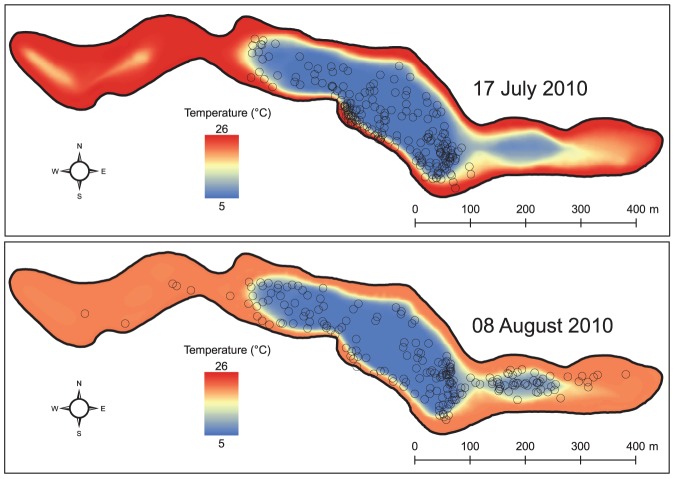
Example of brook trout spatial distribution in relation to the bottom thermal regime of Lake Ledoux. The thermal regimes of the lake bottom were interpolated from the thermal profiles recorded by thermographs in a warm (17 July 2010) and a cold (08 August 2010) period. Open circles represent the radio tracking data collected during four days around the day of the thermal profile. The same fish are represented for both periods.

The average horizontal distances travelled by individuals of both the tactics were lower when the mean daily epilimnion temperature was above 22.4°C (Fig. 4). This result is consistent with the fact that brook charr feeding forays involve both vertical and horizontal movements [21],[49]. When the feeding zones are not accessible due to high water temperatures, a decrease in feeding activity must therefore involve a reduction in both vertical and horizontal movements [19]. By showing that individuals avoid epilimnetic habitats when surface temperature was above 22.4°C, our results show that the thermal regime of the lake could strongly reduce the spatial distribution of brook trout (see an example in Fig. 6). Taken together, the results show that the patterns of horizontal and vertical movements can be decoupled and give complementary information: individuals from the I/IV tactic showed no clear diel pattern with respect to the horizontal distances travelled, whereas those of the II/III tactic increased the extent of their horizontal movements during the night. This latter activity pattern has been documented in other salmonids (e.g. [50]).

### Selected Temperatures

The lake's thermal conditions were not only correlated with the daily movements of individuals but also to their selected temperatures. When the mean daily epilimnion temperature stayed under the 22.4°C threshold, the mean body temperature of individuals closely matched the species optimal temperature for growth [48] (Fig. 5). However, once this threshold was reached, individuals changed their position in the water column to seek cooler areas, decreasing their mean body temperature by approximately 3°C. Javaid and Anderson [51] showed that a change in selected temperature occurred when brook charr, Atlantic salmon, and rainbow trout were starved. For example, brook charr responded with a decrease of about 3°C in their selected temperature within 24 hours after cessation of feeding; selected temperature then rose by 1.5°C and remained constant during the rest of the starvation period (19 days). Within 24 hours after resumption of feeding, their selected temperature returned to its pre-starvation value [51]. The same phenomenon could have occurred in Lake Ledoux during our study. When the mean daily epilimnion temperature was over 22.4°C, fish may not have had access to their primary food source and thus selected lower temperatures to reduce their energy expenditure. This is also in agreement with the above interpretation that forays were favoured when thermal differences between the epilimnion and metalimnion were reduced.

Fish displaying the II/III tactic exhibited lower variability in their selected temperatures than did those with the I/IV tactic (Fig. 5). A closer examination of individual temperature time series (Fig. S2a) suggests two different diel patterns among individuals of the I/IV tactic: half of them remained in warmer water during the day and in cooler water during the night while the other half displayed the reverse pattern. Such an inversion in the phases of individuals can explain the relatively large variability in daily selected temperature and the absence of a daily pattern (Fig. 5). Individuals selecting warmer water during the day tended to show an inverse diel vertical migration (DVM); this was the case with most fish in 2003 and 2005 [1]. DVM is a well-documented behaviour in fish, especially in salmonids (e.g. [8],[52],[53]). These migrations usually involve a selection of lower temperatures during daytime in order to reduce losses due to basal metabolism and conserve energy when food is limited [8],[47]. In Lake Ledoux, as least 50% of the individuals exhibited the opposite behaviour, which was also observed by Bertolo et al. [1]. Although this behaviour was explained by an optimization of their energy budget [1], it is difficult to explain why it is not adopted by all individuals.

DVM is generally considered a characteristic of the entire population [52],[53]. However, recent studies have shown that different individual traits such as sex, size, or condition can induce different DVM strategies within a population [54],[55]. Hight and Lowe [56] showed that only female leopard sharks (*Triakis semifasciata*) performed inverse DVM, which likely increases physiological functions involved in reproduction. Mehner and Kasprzak [57] found the existence of partial DVM in a lake inhabited by two cisco species, the common vendace (*Coregonus albula*) and the Fontane cisco (*Coregonus fontanae*): both species performed DVM, but depending on fish size, water temperature, and feeding rates in the daytime habitat, a varying proportion of individuals remained all day in the same habitat. In our study, no such difference in sex, body size, or condition was found either between the tactics or between the two types of diel patterns within the I/IV tactic (results not shown). It is possible that other traits such as social status [58] or behavioural syndromes [59] may have contributed to the occurrence of these opposite behaviours. The existence of different diel thermal behaviours could also be explained by variable cost–benefit ratios resulting from interactions between feeding gain and bioenergetics efficiency [7],[19],[57]. For instance, it could be more profitable to perform a direct DVM when food is limited in order to save energy whereas it could be more profitable to switch to an inverse DVM when food is unlimited, in order to optimize the digestion process and thus maximize growth efficiency. It is also possible that brook charr follow the distribution of their prey, which could exhibit direct or inverse DVM depending on habitat type, as shown for basking shark *Cetorhinus maximus* [60].

### Conclusion

Our study confirms the interannual persistence of the thermoregulatory tactics in brook charr observed by Bertolo et al. [1] and thus the sustained spatio-temporal segregation of individuals over the years, presumably for a better exploitation of available resources [61]. However, our results also showed that the expression of these tactics can be strongly influenced by the lake's thermal regime (e.g., Fig. 6). Mathematical models predict that the surface temperature of Canadian Shield lakes will increase with global warming [62],[63]. These results therefore provide insight into the potential impact of global warming on cold-water species by reducing the accessibility of resources and the expression of thermal tactics.

## Supporting Information

Figure S1 **Time series of hourly temperature in Lake Ledoux from 5 July to 29 August 2010.** Black and grey lines represent measurements taken at 1 m intervals starting from 0 m and 0.5 m in depth, respectively. (PDF)

Figure S2 **Individual thermal patterns for the I/IV (a) and II/III (b) tactics during the period considered in the thermal tactics analyses (5–23 July 2010; day of the year 186–204).** Black dots represent the observed body temperatures and lines represent the values predicted by AEM modelling. Each fish is identified by its transmitter number. For each individual, the number of data (n), the percentage of missing values (% mv), the mean adjusted coefficient of determination (R_adj_^2^), and the mean body temperature are indicated. (PDF)
