# Supplementary figures and images for: Effects Of Lake Warming On Behavioural Thermoregulatory Tactics In A Cold-Water Stenothermic Fish
Source: PLoS One. 2014 Mar 24;9(3):e92514. doi: 10.1371/journal.pone.0092514 (PMC3963910; doi:10.1371/journal.pone.0092514)

Temperature (°C)

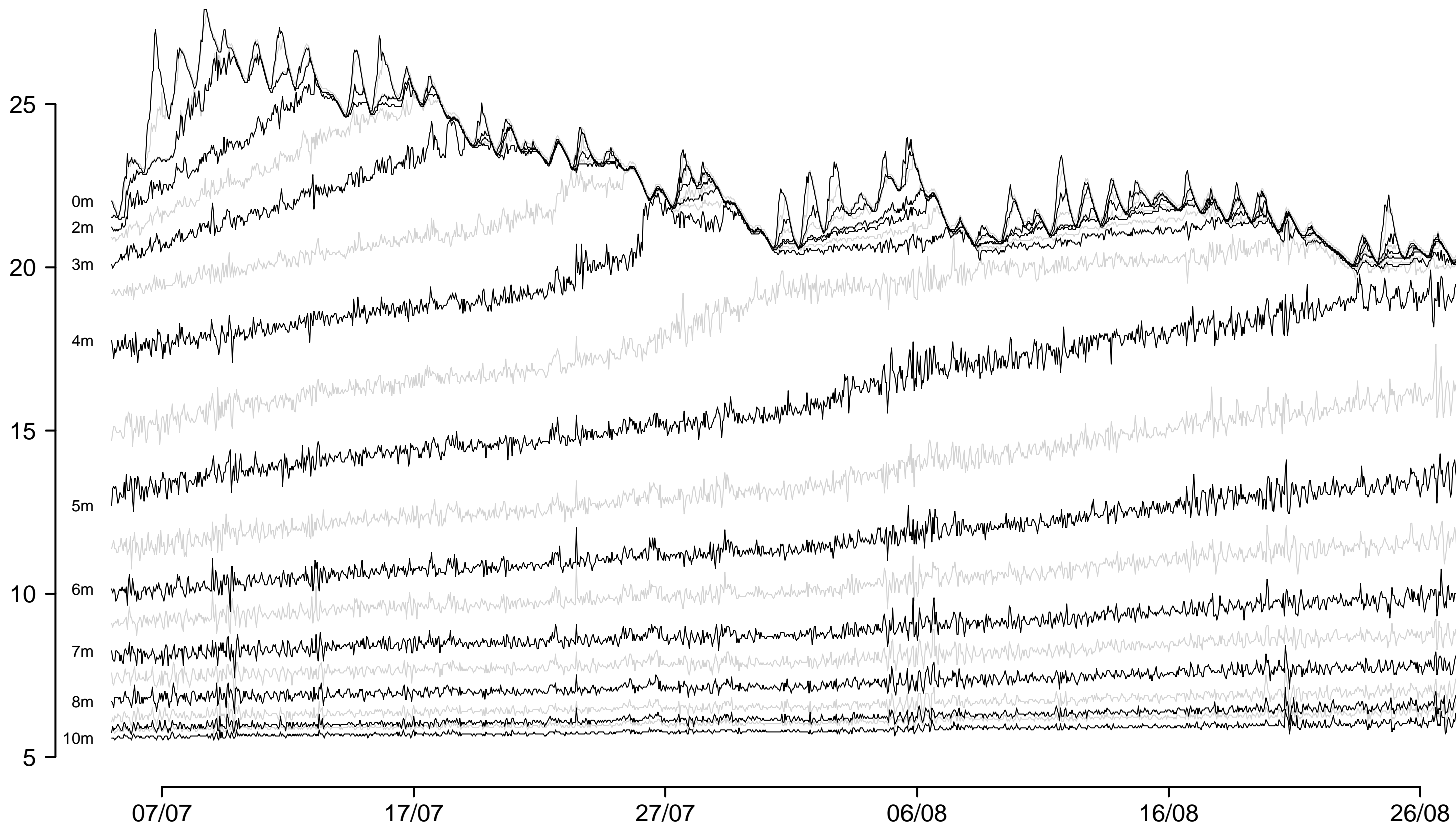

Supplement: Figure S1 — (PDF) [file pone.0092514.s001.pdf]
