# Supplementary material for: Effects Of Lake Warming On Behavioural Thermoregulatory Tactics In A Cold-Water Stenothermic Fish
Source: PLoS One. 2014 Mar 24;9(3):e92514. doi: 10.1371/journal.pone.0092514 (PMC3963910; doi:10.1371/journal.pone.0092514)
Supplement: Figure S2 — (PDF) [file pone.0092514.s002.pdf]

Figure S2a. Individual thermal patterns for the diel tactics

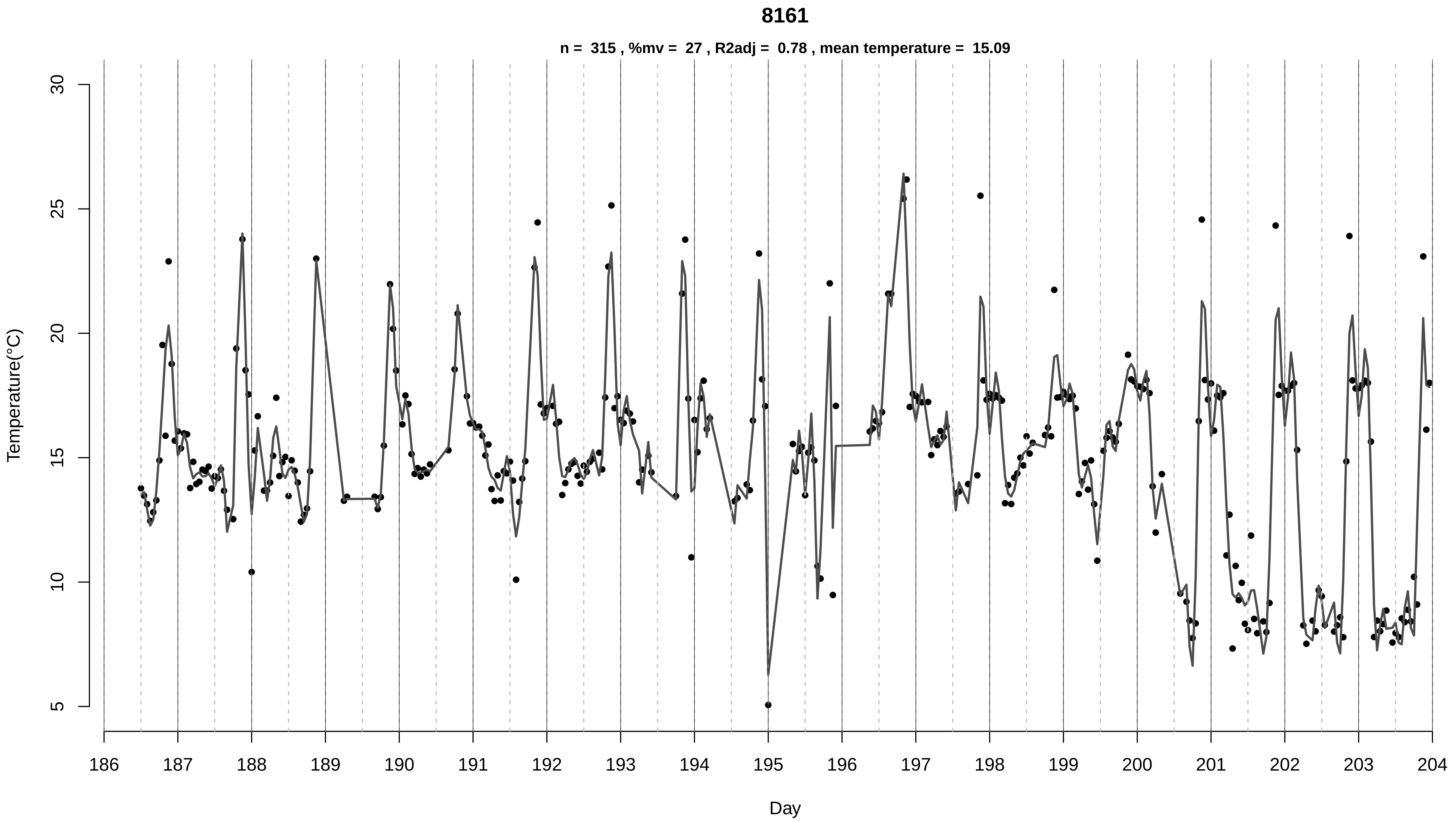

8161
n = 315 , %mv = 27 , R2adj = 0.78 , mean temperature = 15.09
Temperature(°C)
5
10
15
20
25
30
186
187
188
189
190
191
192
193
194
195
196
197
198
199
200
201
202
203
204
Day

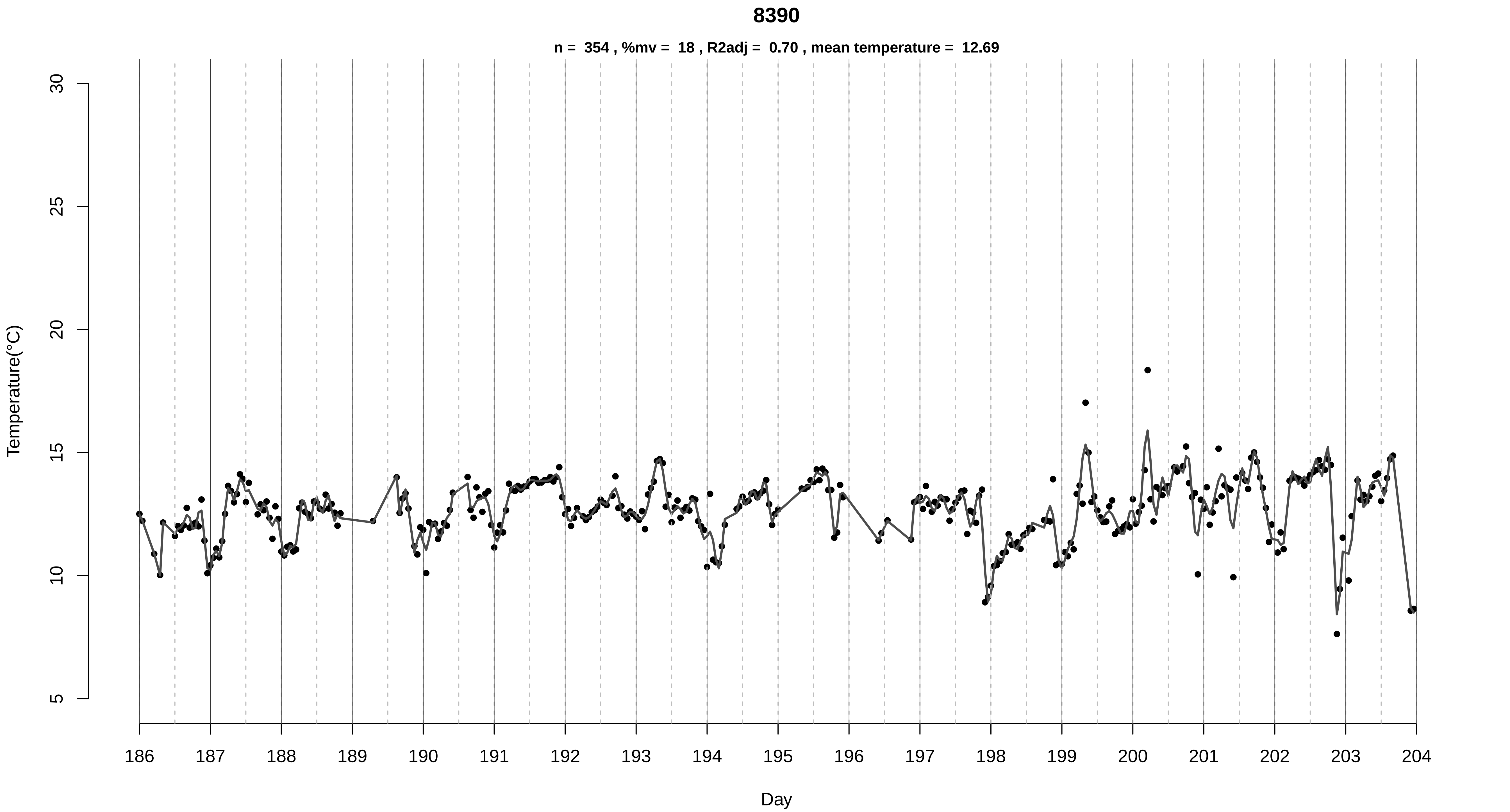
8390
n = 354 , %mv = 18 , R2adj = 0.70 , mean temperature = 12.69
Temperature(°C)
5
10
15
20
25
30
186
187
188
189
190
191
192
193
194
195
196
197
198
199
200
201
202
203
204
Day

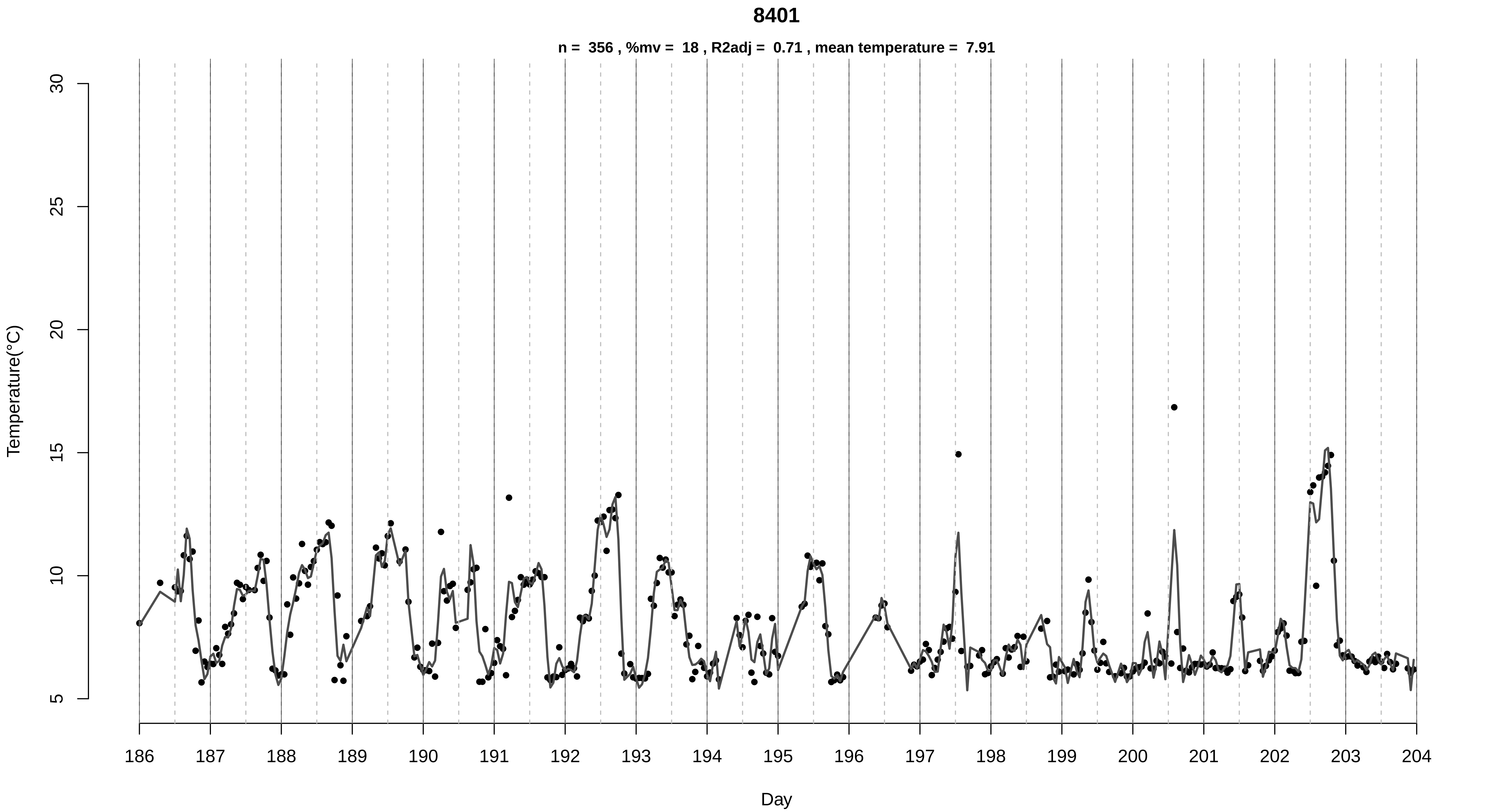
8401
n = 356 , %mv = 18 , R2adj = 0.71 , mean temperature = 7.91
Temperature(°C)
5
10
15
20
25
30
186
187
188
189
190
191
192
193
194
195
196
197
198
199
200
201
202
203
204
Day

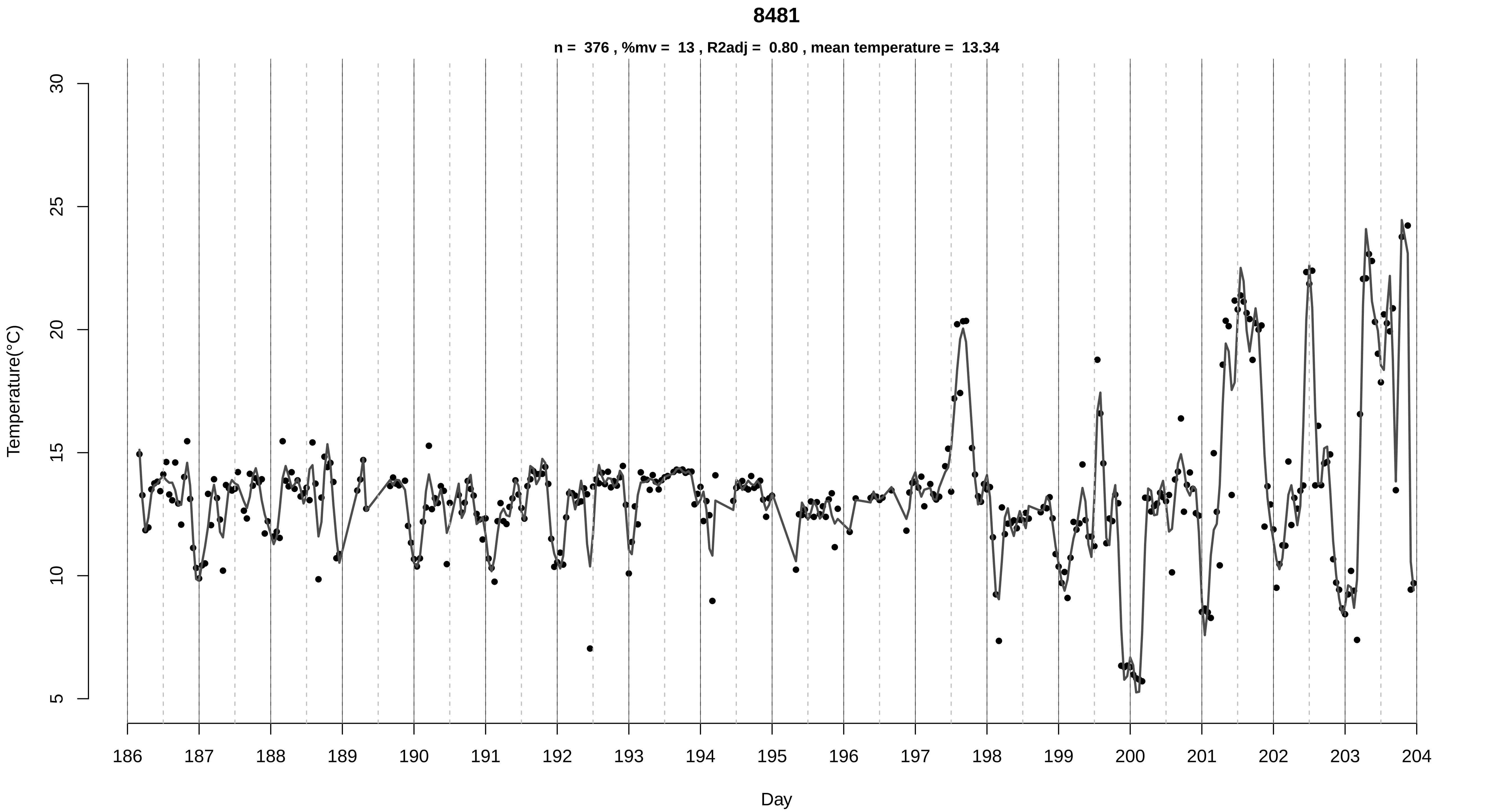

8481
n = 376 , %mv = 13 , R2adj = 0.80 , mean temperature = 13.34
Temperature(°C)
5
10
15
20
25
30
186
187
188
189
190
191
192
193
194
195
196
197
198
199
200
201
202
203
204
Day

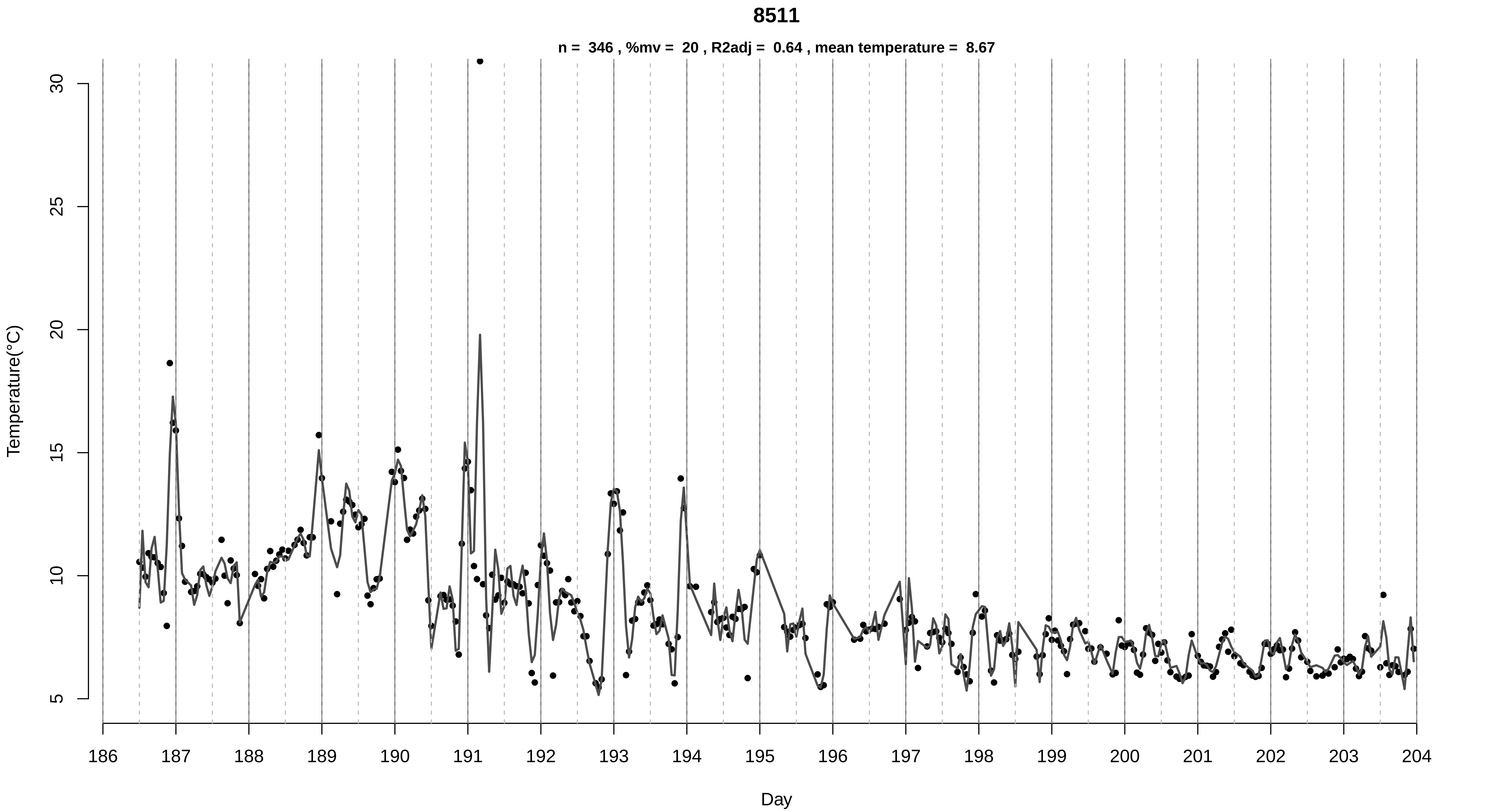

8511
n = 346 , %mv = 20 , R2adj = 0.64 , mean temperature = 8.67
Temperature(°C)
5
10
15
20
25
30
186
187
188
189
190
191
192
193
194
195
196
197
198
199
200
201
202
203
204
Day

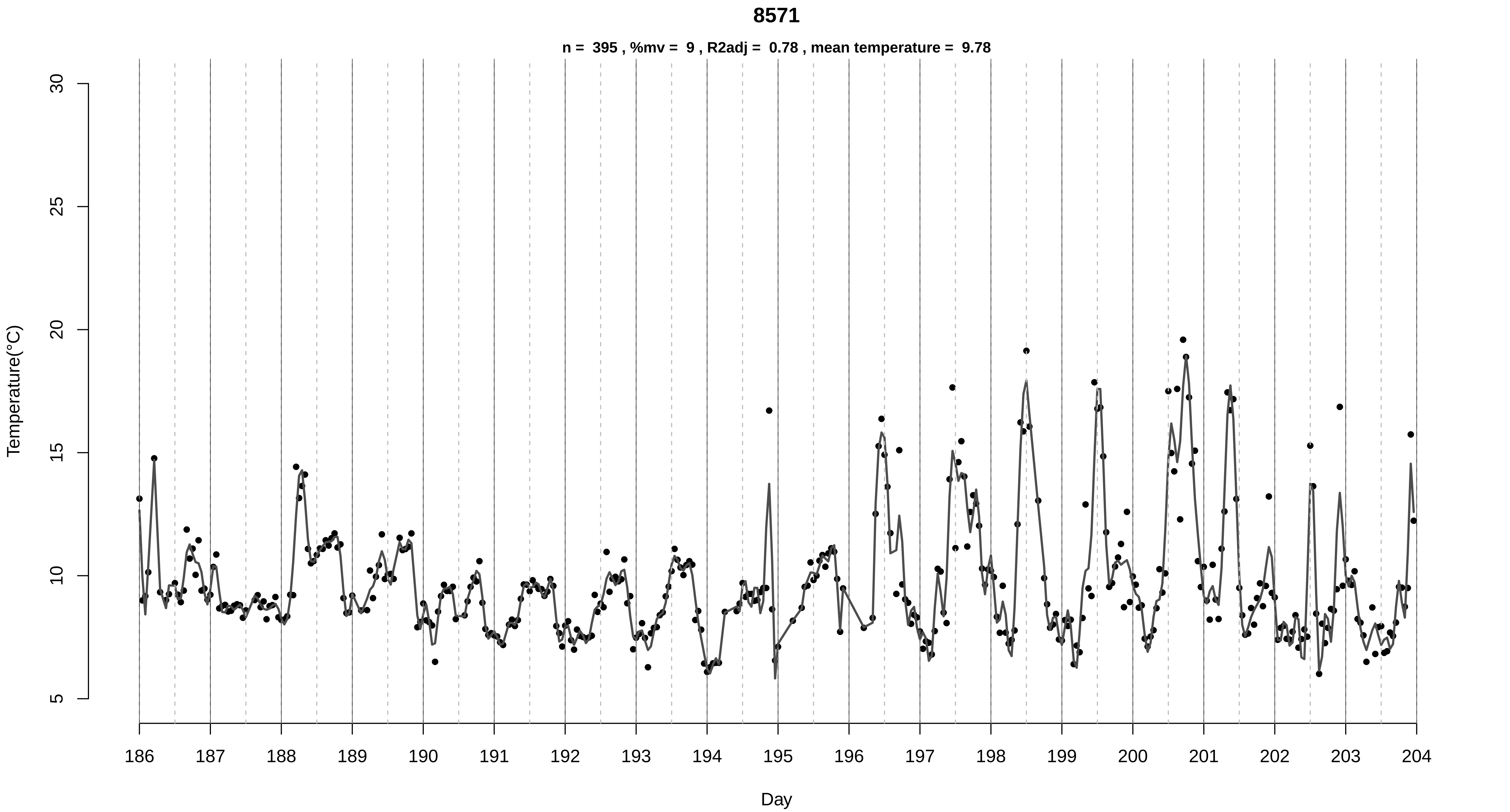

8571
n = 395 , %mv = 9 , R2adj = 0.78 , mean temperature = 9.78
Temperature(°C)
30
25
20
15
10
5
186
187
188
189
190
191
192
193
194
195
196
197
198
199
200
201
202
203
204
Day

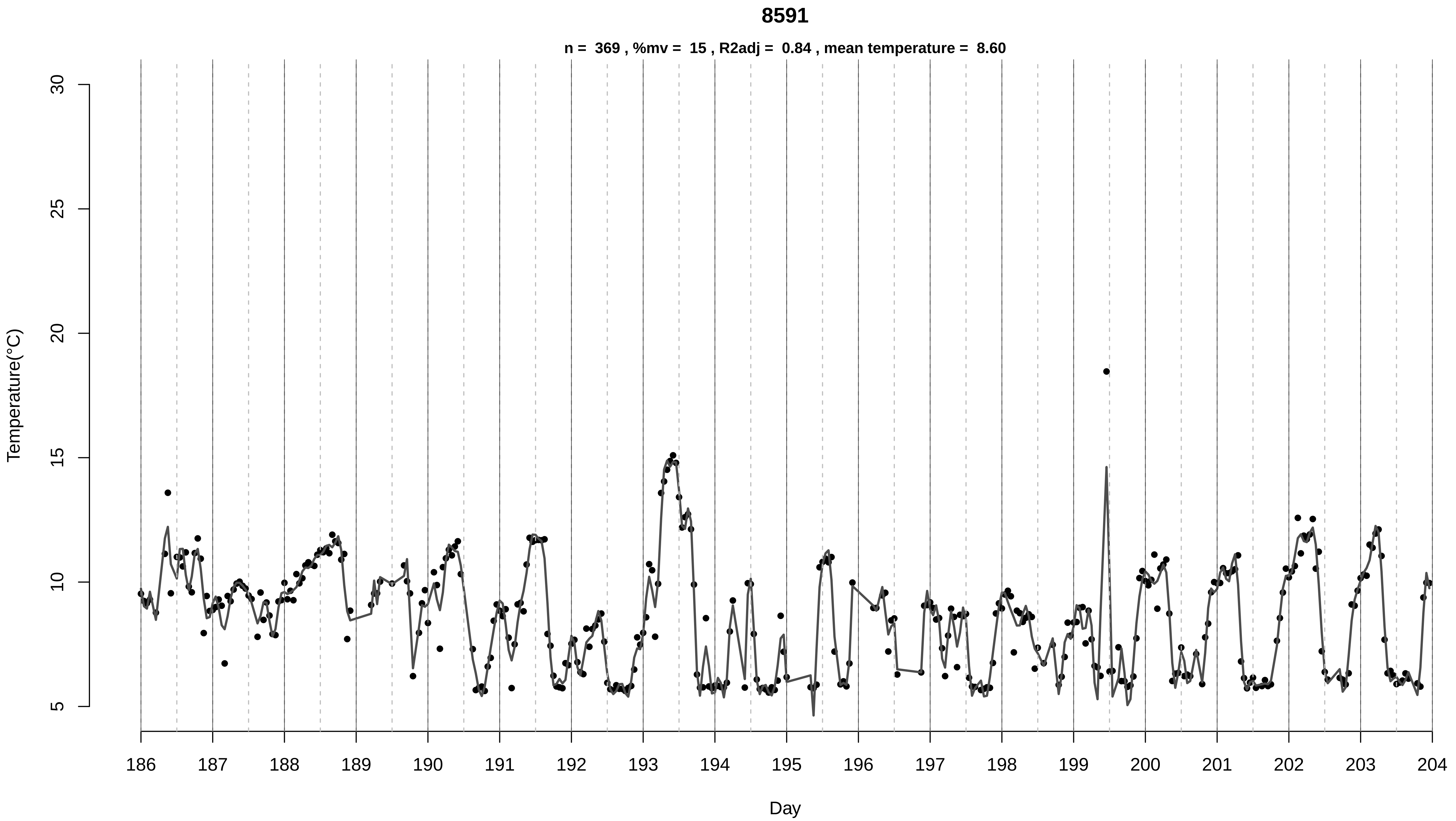

8591
n = 369 , %mv = 15 , R2adj = 0.84 , mean temperature = 8.60
Temperature(°C)
30
25
20
15
10
5
186
187
188
189
190
191
192
193
194
195
196
197
198
199
200
201
202
203
204
Day

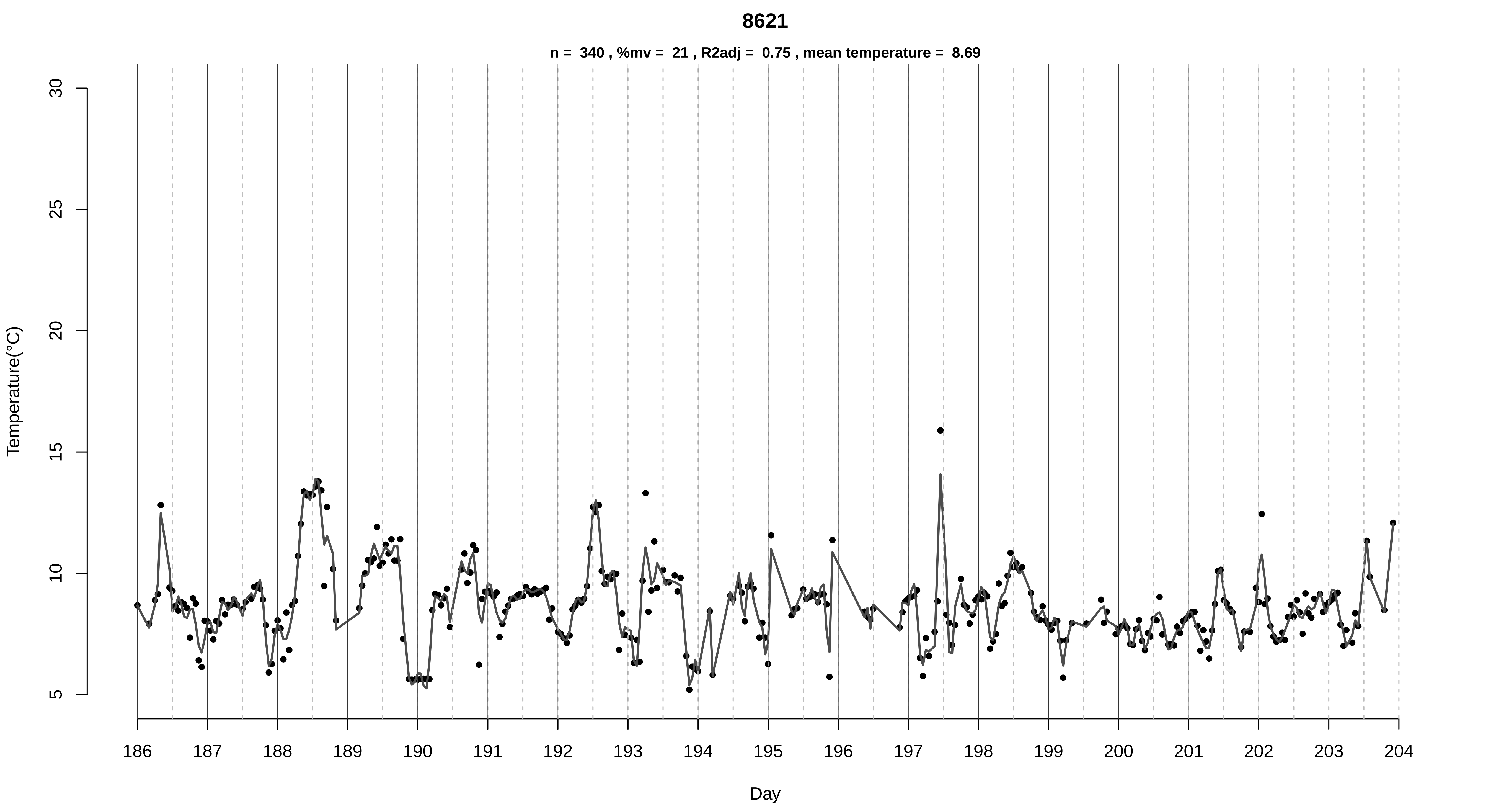

8621
n = 340 , %mv = 21 , R2adj = 0.75 , mean temperature = 8.69
Temperature(°C)
5
10
15
20
25
30
186
187
188
189
190
191
192
193
194
195
196
197
198
199
200
201
202
203
204
Day

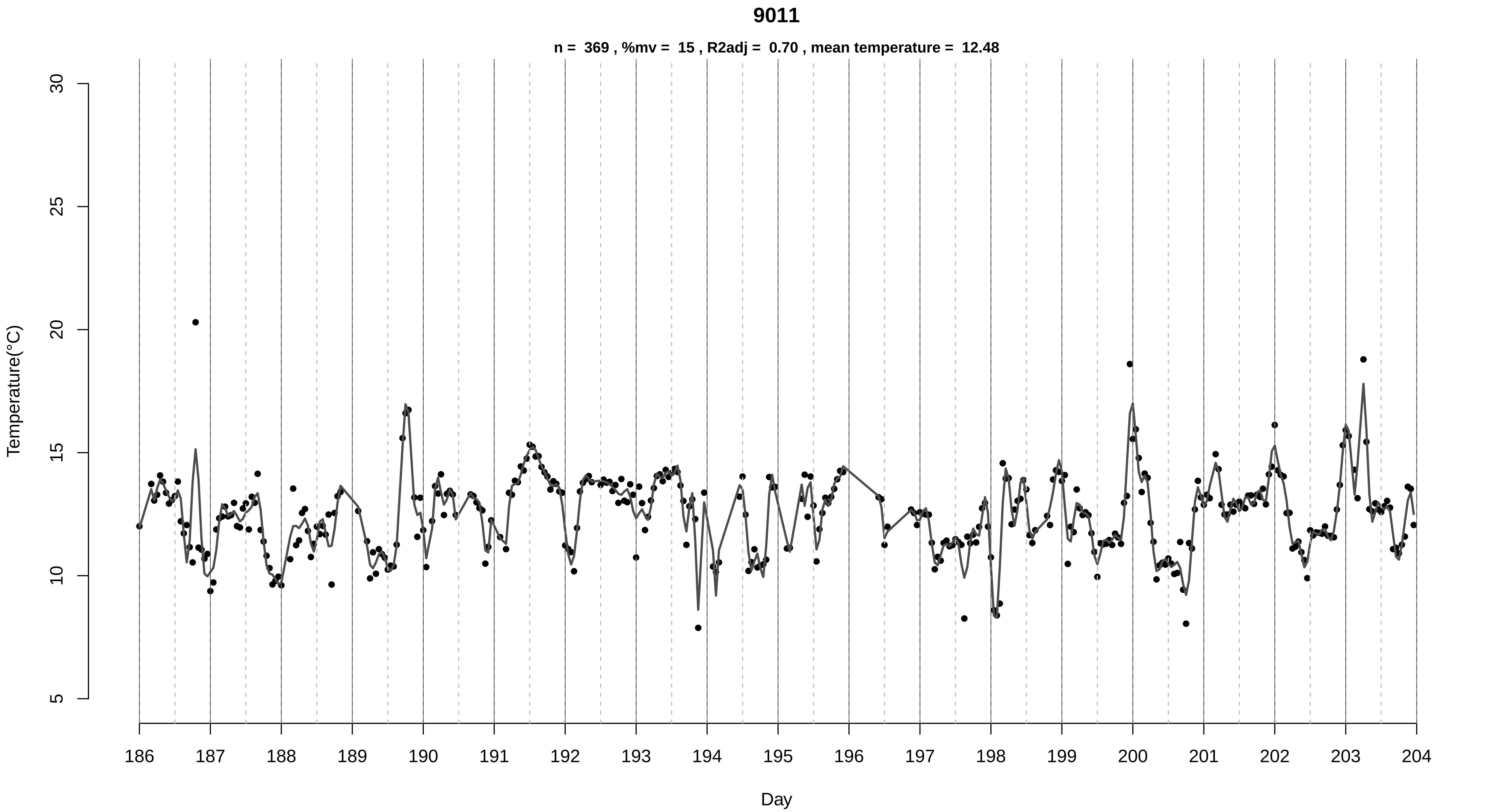

9011
n = 369 , %mv = 15 , R2adj = 0.70 , mean temperature = 12.48
Temperature(°C)
5
10
15
20
25
30
Day
186
187
188
189
190
191
192
193
194
195
196
197
198
199
200
201
202
203
204

Figure S2b. Individual thermal patterns for the broad tactics

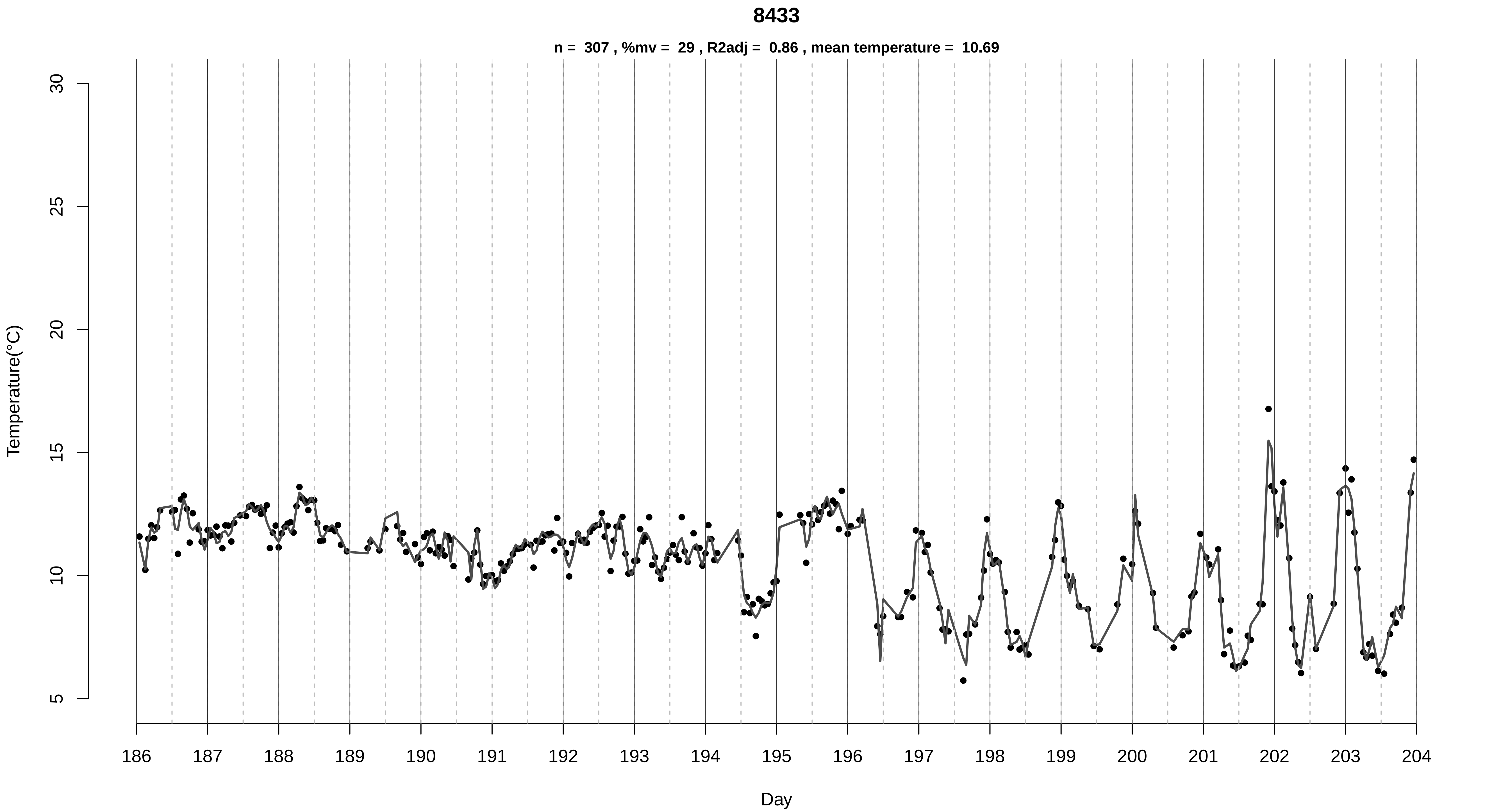
8433
n = 307 , %mv = 29 , R2adj = 0.86 , mean temperature = 10.69
Temperature(°C)
5
10
15
20
25
30
186
187
188
189
190
191
192
193
194
195
196
197
198
199
200
201
202
203
204
Day

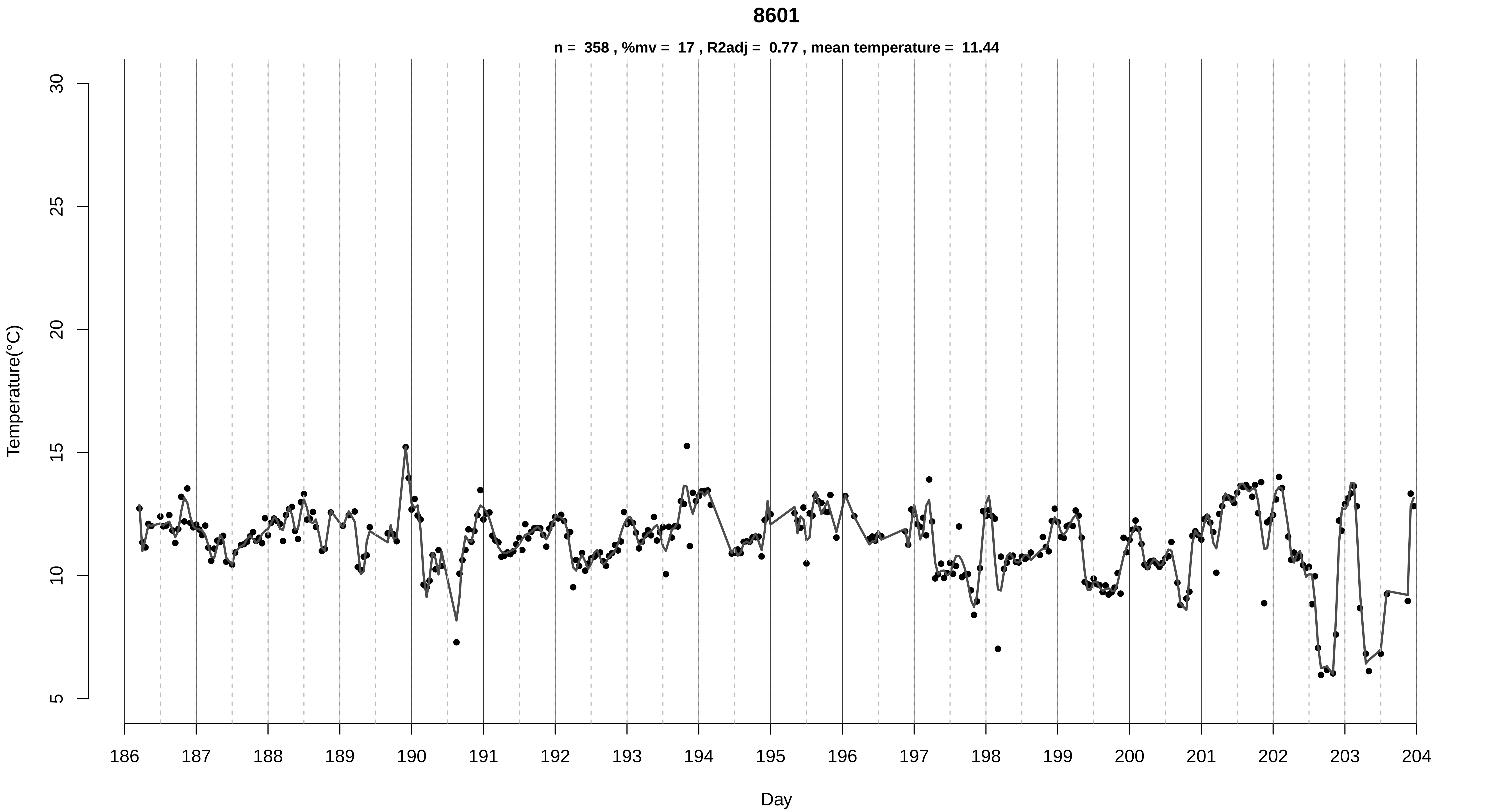
8601
n = 358 , %mv = 17 , R2adj = 0.77 , mean temperature = 11.44
Temperature(°C)
Day
5
10
15
20
25
30
186
187
188
189
190
191
192
193
194
195
196
197
198
199
200
201
202
203
204

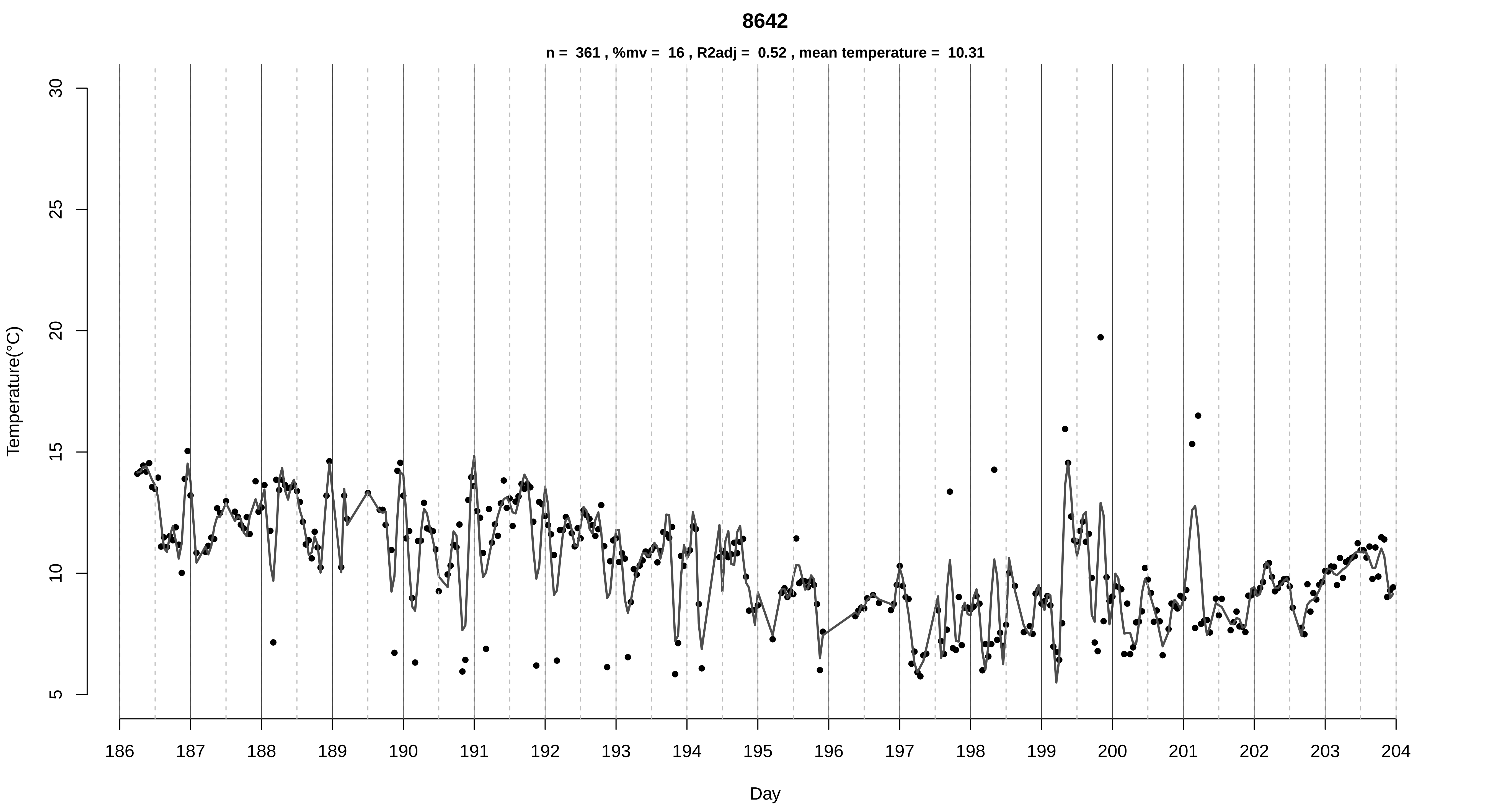
8642
n = 361 , %mv = 16 , R2adj = 0.52 , mean temperature = 10.31
Temperature(°C)
5
10
15
20
25
30
186
187
188
189
190
191
192
193
194
195
196
197
198
199
200
201
202
203
204
Day

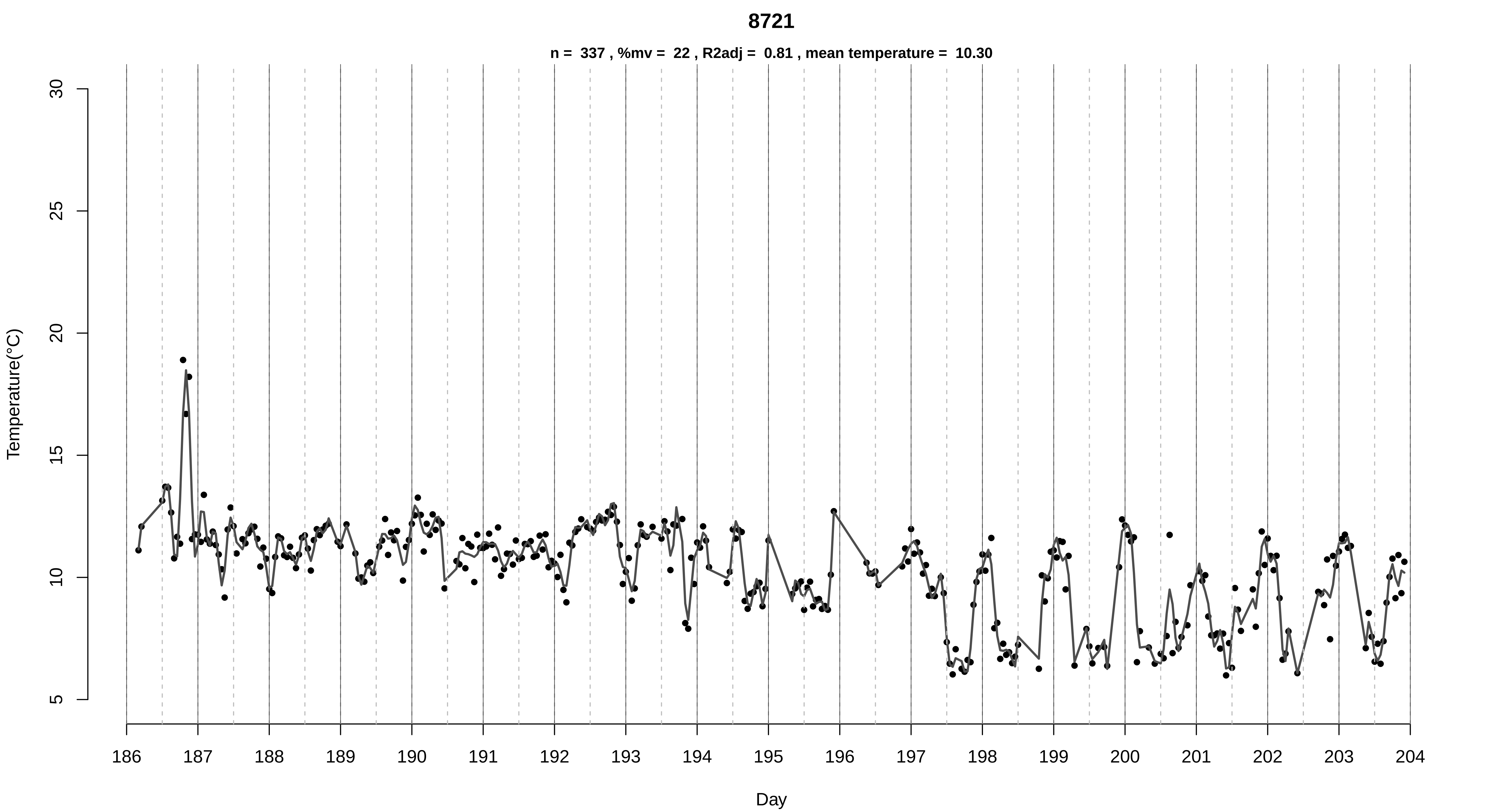

8721
n = 337 , %mv = 22 , R2adj = 0.81 , mean temperature = 10.30
Temperature(°C)
5
10
15
20
25
30
186
187
188
189
190
191
192
193
194
195
196
197
198
199
200
201
202
203
204
Day

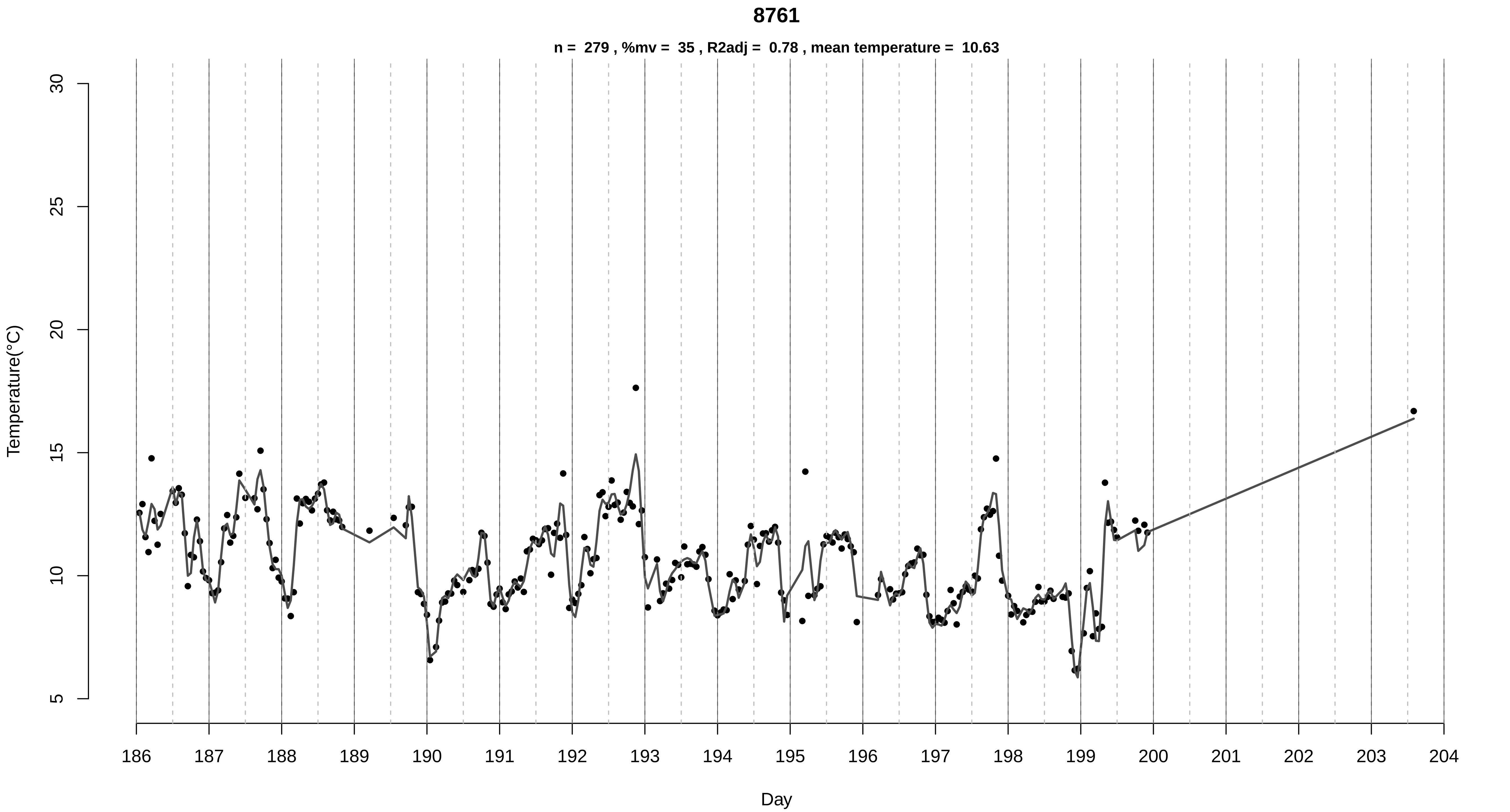

8761
n = 279 , %mv = 35 , R2adj = 0.78 , mean temperature = 10.63
Temperature(°C)
5
10
15
20
25
30
186
187
188
189
190
191
192
193
194
195
196
197
198
199
200
201
202
203
204
Day

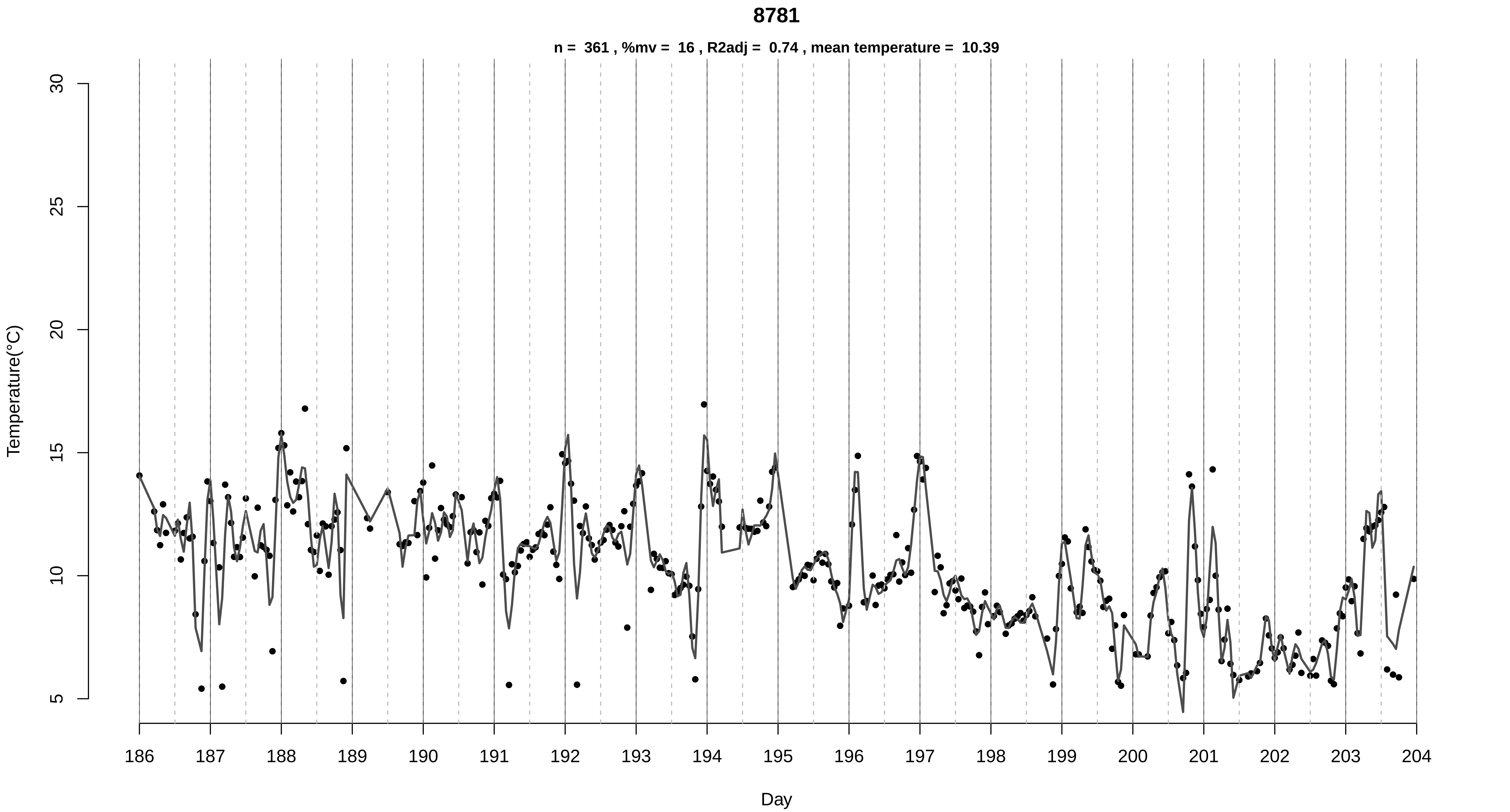

8781
n = 361 , %mv = 16 , R2adj = 0.74 , mean temperature = 10.39
Temperature(°C)
5
10
15
20
25
30
186
187
188
189
190
191
192
193
194
195
196
197
198
199
200
201
202
203
204
Day

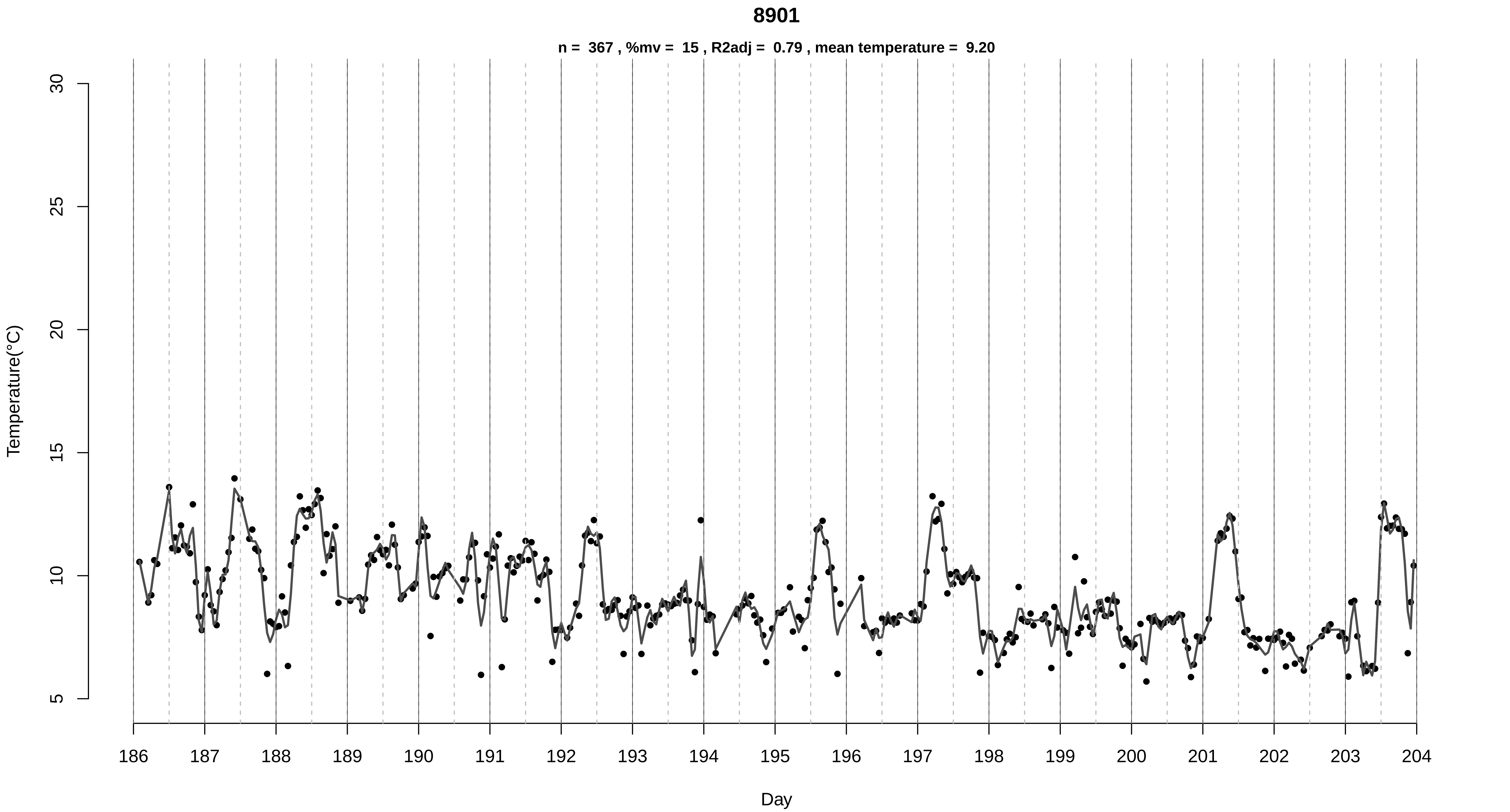
8901
n = 367 , %mv = 15 , R2adj = 0.79 , mean temperature = 9.20
Temperature(°C)
5
10
15
20
25
30
186
187
188
189
190
191
192
193
194
195
196
197
198
199
200
201
202
203
204
Day
